# An extended cost-effectiveness analysis of decentralised TB diagnostic testing with Molbio Truenat MTB/RIF versus hub-and-spoke GeneXpert MTB/RIF in Mozambique and Tanzania

**DOI:** 10.1136/bmjgh-2025-020117

**Published:** 2025-11-19

**Authors:** Saima Bashir, Lelisa Fekadu Assebe, Akash Malhotra, Délio Elísio, Antonio Machiana, Anange Lwilla, Jerry Hella, Neenah Young, Mikaela Watson, Vinzeigh Leukes, Adam Penn-Nicholson, Morten Ruhwald, Leyla Larsson, David Dowdy, Manuela De Allegri, Claudia M Denkinger

**Affiliations:** 1 Department of Infectious Disease and Tropical Medicine, Heidelberg University Hospital, Heidelberg, Germany; 2 University of Manchester, Manchester, UK; 3 Global Public Health and Primary Care Medicine, University of Bergen, Bergen, Norway; 4 Disease Prevention and Control, Ethiopia Ministry of Health, Addis Ababa, Ethiopia; 5 Department of Global Health, University of Washington, Seattle, Washington, USA; 6 Department of Epidemiology, Johns Hopkins University Bloomberg School of Public Health, Baltimore, Maryland, USA; 7 Centro de Investigação em Saúde de Manhiça Manhiça, Maputo, Mozambique; 8 Instituto Nacional de Saúde (INS), Marracuene, Mozambique; 9 National Institute for Medical Research, Dar es Salaam, Tanzania; 10 Tuberculosis Research Group, Ifakara Health Institute, Ifakara, Tanzania; 11 FIND, Geneva, Switzerland; 12 Infectious Diseases and Tropical Medicine, Ludwig Maximilians University Munich, Munchen, Germany; 13 Biomedical Research and Training Institute, Harare, Zimbabwe; 14 Department of International Health, Bloomberg School of Public Health, The Johns Hopkins University School of Medicine, Baltimore, Maryland, USA; 15 Heidelberg Institute of Global Health, Heidelberg University, Heidelberg, Germany; 16 Center of Infectious Disease, Heidelberg University, Heidelberg, Germany; 17 German Center for Infection Research (DZIF), Heidelberg, Germany

**Keywords:** Tuberculosis, Health economics

## Abstract

**Background:**

In low- and middle-income countries (LMICs), tuberculosis (TB) regained its status as the leading cause of death from a single infectious agent in 2023, surpassing COVID-19. Rapid and accurate diagnosis is critical, with decentralised diagnostic strategies offering a promising solution. Although decentralised diagnostic strategies have been proven cost-effective, further evidence is needed on affordability and equity in high-burden settings. This study, part of a multicentre real-world cluster-randomised controlled trial (cRCT), assessed implementation costs and out-of-pocket (OOP) expenditures across socioeconomic status (SES) groups from a societal perspective.

**Methods:**

The TB-CAPT Core trial compared decentralised point-of-care TB testing using the Molbio Truenat platform (intervention) with the hub-and-spoke Xpert MTB/RIF Ultra model (control) in Tanzania and Mozambique. Economic data were collected as part of the trial along with asset ownership information using an equity tool. Multiple correspondence analysis was used to construct an asset index for each country. Extended cost-effectiveness analysis estimated incremental participant costs for TB diagnosis and health outcomes (number of participants who initiated treatment within seven and sixty days) across SES groups. Distributional cost-effectiveness analysis assessed facility-based diagnostic cost per treatment initiated from a societal perspective across SES groups. Regression analyses explored the intervention’s impact on direct, indirect and total costs.

**Results:**

Average OOP expenditures were lower in the intervention arm (US$8.82) than in the control group (US$13.61). Regression analysis confirmed a significant cost reduction. Least poor participants experienced greater cost-savings (−US$6.36 vs −US$2.93), while the poorest had a higher number of TB treatment initiations within 7 days of diagnosis (poorest vs least poor: 28 vs 8). The incremental cost-effectiveness ratio for the poorest group was US$778, whereas for the other two groups, the intervention showed higher treatment initiation (52 vs 36 for middle, 33 vs 25 for least poor) at lower costs than the standard of care.

**Conclusion:**

The intervention reduced patient costs and improved outcomes across SES groups. Decentralised TB testing with the Molbio Truenat platform is both cost-saving and more effective and cost-effective compared with a hub-and-spoke model in Mozambique and Tanzania.

**WHAT IS ALREADY KNOWN ON THIS TOPIC**

- Tuberculosis (TB) remains a major cause of death in low- and middle-income countries; timely and accurate diagnosis is essential for effective management and transmission control.
- Molecular diagnostic tools such as Xpert MTB/RIF Ultra are widely used; their dependence on centralised hub-and-spoke networks can introduce diagnostic delays, postponing treatment initiation.
- Decentralised diagnostics such as Truenat may alleviate access constraints, but real-world evidence on affordability, equity impacts across socioeconomic strata and patient out-of-pocket costs, despite apparently free public TB services, remains limited.

**WHAT THIS STUDY ADDS**

- This is the first real-world study using extended cost-effectiveness analysis and distributional cost-effectiveness analysis to evaluate equity and financial outcomes of decentralised TB diagnostics.
- Truenat reduced average out-of-pocket costs and improved early treatment initiation, particularly benefitting the poorest participants.
- Least poor patients saved more in absolute costs, while the poorest groups experienced greater health gains.

**HOW THIS STUDY MIGHT AFFECT RESEARCH, PRACTICE OR POLICY**

- By demonstrating both financial and equity benefits, this study strengthens the case for scaling up decentralised TB diagnostic services in high-burden settings.
- Policymakers can use this evidence to justify investments in point-of-care molecular diagnostics not just for their effectiveness but also for their role in advancing equity and reducing catastrophic patient costs.
- The methods used provide a framework for evaluating health interventions through both economic and distributional lenses, encouraging future research to integrate equity-focused evaluations into cost-effectiveness studies.

## Background

 Tuberculosis (TB) resumed its position as the leading cause of death from a single infectious agent, surpassing COVID-19 in 2023. Globally, the estimated number of TB cases was 10.8 million in 2023, with approximately 1.25 million deaths.^1^ Effective TB management relies on a rapid and accurate diagnosis to enable prompt initiation of a treatment regimen, reducing losses to follow-up.^2^

Molecular testing, such as Cepheid Xpert MTB/RIF Ultra (Xpert; Sunnyvale, CA, USA), is globally recommended as the standard of care (SOC) for TB diagnosis. However, in many high-burden settings, onsite testing at peripheral health centres is often still limited to the less sensitive sputum smear microscopy (SSM) because of infrastructure requirements and cost of molecular testing. To enhance access to more sensitive molecular testing, many high-burden countries use a hub-and-spoke model, for which sputum samples are collected onsite and sent to centralised facilities for Xpert testing with results then being sent back to peripheral health units.^3^,^5^ Although Xpert has been deployed at lower levels of the health system in some settings, it typically requires stable electricity, temperature control and a laboratory environment, which limit its use in peripheral or rural settings. However, more accurate onsite testing as compared with smear in decentralised settings has become feasible with the introduction of new molecular tests, such as the Molbio Truenat MTB/RIF platform (‘Molbio’; Molbio Diagnostics, Verna, India) and MTB Plus and MTB-RIF Dx assays.

These diagnostic advancements raise new questions about pursuing the most effective and cost-effective testing strategy. Using a pragmatic design, the TB-CAPT CORE trial evaluated the effectiveness of the Truenat platform and/or MTB assays (‘Truenat’) on enhancing the TB diagnostic pathway at primary health clinics in Mozambique and Tanzania.^6^ In both Mozambique and Tanzania, 97.2% (125/129) of participants with microbiologically confirmed pulmonary TB in the intervention arm initiated TB treatment within 7 days of enrolment, compared with 63% (96/152) in the SOC arm.^6 7^ As part of the TB-CAPT CORE trial, the accompanying cost-effectiveness analysis (CEA) estimated an incremental cost per patient with TB initiated on treatment at US$403 (−US$103, US$941) in Mozambique and US$580 (US$167, US$1638) in Tanzania, demonstrating that decentralised TB testing with Molbio Truenat is cost-effective compared with the hub-and-spoke testing model in both countries.

To inform decision-making at the national programme level, evidence is also required on affordability, equity and financial risk protection. Despite free TB diagnosis and treatment within public health systems, many patients with TB and their families continue to bear considerable direct and indirect costs associated with illness and care-seeking. In low- and middle-income countries (LMICs), these expenses can amount to nearly half of an affected household’s annual income, posing significant barriers to healthcare access and increasing the likelihood of financial hardship or further impoverishment. These poverty-exacerbating effects are most pronounced among the lowest socioeconomic status (SES) groups, for whom the consequences of TB are especially severe.^8^,^12^ While most available evidence is drawn from multi-country systematic reviews or studies in other LMICs, there is limited published data specifically on the economic burden of TB care in Mozambique and Tanzania. To our knowledge, no nationally representative studies from these countries have comprehensively quantified the direct and indirect costs borne by patients with TB. This gap underscores the need for setting-specific evidence on financial barriers to TB care. These expenses can lead to delayed care-seeking and increased loss to follow-up, which may further contribute to the spread of disease.^13^,^16^

This extended cost-effectiveness analysis (ECEA) complements evidence emerging from the effectiveness and cost-effectiveness assessments by exploring how the benefits and financial burdens of a decentralised TB diagnostic intervention are distributed across SES groups. We focus on the equity and financial implications by examining both direct (medical and non-medical) and indirect (working days lost due to illness and clinic stay) out-of-pocket (OOP) expenditures incurred by participants. To further understand the intervention’s impact, we apply a distributional cost-effectiveness analysis (DCEA) comparing the decentralised model with SOC, evaluating the incremental facility-based diagnostic cost per participant initiating TB treatment within 7 and 60 days from societal perspective for each SES group.

## Methods

### Study design and participants

The TB-CAPT Core trial is a pragmatic, cluster-randomised controlled trial (cRCT) that was conducted at 29 peripheral health facilities located across four participating institutions in Tanzania and Mozambique between August 2022 and June 2023. Out of 29 facilities, 15 were included in the intervention group, where participants with presumed TB were tested onsite using Truenat. The SOC, as implemented in 14 healthcare facilities, consisted of a hub-and-spoke model that included Xpert testing off site or SSM on site followed by confirming Xpert testing off site. In the intervention arm, a single visit was sufficient for most participants with microbiologically confirmed pulmonary TB, as 125 out of 152 participants (82.2%) initiated treatment on the same day of presentation. In contrast, only 5 out of 150 participants (3.3%) in the control clinics started treatment on the same day. The trial’s protocol and findings have been published elsewhere.^6^

### Outcome measurement

We combined data from both countries to perform a pooled analysis, as individual country sample sizes were limited. We estimated the distribution of OOP expenditures and primary and secondary health outcomes of the trial including treatment initiation for the disease within 7 and 60 days of enrolment across SES groups, comparing the intervention and SOC arms for both countries. We also examined the incremental facility-based diagnostic cost per participant who started TB treatment within 7 and 60 days of enrolment, adopting a societal perspective and analysing variations across different SES groups.

Our primary ECEA outcome was the incremental per-participant OOP expenditure averted from initiating treatment within 7 days of enrolment. The secondary outcome was the incremental OOP expenditures per incremental microbiologically positive participant receiving treatment within 60 days of enrolment. In DCEA, we also analysed the incremental facility-based diagnostic cost per participant who initiated TB treatment within 7 and 60 days, taking a societal perspective and examining variations across different SES groups. Facility-based diagnostic cost includes both OOP expenditures such as transportation, food and lost income, etc, and health system costs related to the provision of diagnostic service. Health outcomes, including confirmed TB cases, and the number of participants starting treatment within 7 and 60 days are detailed by country and trial group in online supplemental appendix figure A1. The trial protocol and its results were published earlier.^6^

### Analysis

#### Patient cost data

A total of 5005 people who accessed the study clinics during the study were assessed for eligibility. Of those, 4034 (80.6%) were enrolled in the trial. Primary data were collected using a survey from the participants and caregivers (respondents). A systematic sample of every 10th participant (around 10%) was taken to assess expenditures across the 29 clinics, using blocked randomisation within four strata, to ensure equal representation across countries and regions. Complete participant cost data were collected from 388 patients across both countries and trial groups.

The survey used a standard set of questions to determine participants’ costs, covering both direct and indirect costs related to TB diagnostics. Direct costs included all direct medical costs, such as consultation fees, chest X-rays and other diagnostic tests, as well as OOP expenditures for medications or any other supplies. Direct non-medical costs included participant and caregiver round trip travel expenses, additional food costs (that would not have been spent if they were eating at home or at work), participant and caregiver childcare, and any other related costs. The survey also collected information on the value of any property sold, borrowed money or taken out a loan due to illness, along with work lost because of illness and staying in clinic. However, only work lost due to illness and productivity losses due to clinic stay costs were used in calculating indirect costs. Values related to property sold, loans or borrowed money were excluded as they represent financing strategies rather than actual productivity or time losses due to illness.

#### Health system cost data

In the parallel study on CEA, for the estimation of health system cost per participant, the data were collected from a sample of 19 out of 29 intervention and standard care clinics.^17^ Data collection included TB and HIV testing capacity, sample transport, testing protocols, infrastructure and implementation processes. The data were organised into categories covering equipment, staffing, consumables, training, communication, monitoring and evaluation, and other expenses. Capital assets such as testing equipment were annuitised and depreciated linearly over an anticipated 10-year lifespan at a 3% annual discount rate. Equipment costs were derived from product catalogues and trial expense reports. All the tools used in health system cost collection are provided in online supplemental material S1, including data collection tools and data analysis (online supplemental tables S1 and S2).

#### Patient cost estimation

Both direct and indirect participants’ costs were converted to US dollars using the 2022/2023 current exchange rates (US$1=2505 Tanzanian Shillings, 63.25 Mozambican Metical). Indirect costs were estimated as the opportunity cost of time spent seeking care, from the onset of symptoms to the initiation of treatment. This included the total work missed throughout the illness duration. The indirect cost of lost working days and clinic stay was calculated using the minimum monthly wage (Mozambique: 5800 Mozambican Metical, Tanzania: 140 000 Tanzanian Shillings).^18^ All participants’ cost data were collected during the first visit, covering all previous work lost due to the illness.

The cost of workdays lost was calculated by multiplying the time (such as number of days participants did not work) by the minimum wage per day, with a working month considered to be 26 days (6 days per week). The cost of clinic stay was calculated by converting hours and minutes into the corresponding wage lost (hours: 26*8=208, minutes: 208*60=12 480).^18^

In the intervention group, the decentralised placement and short turn-around time of the tests allowed for both provision of results and the start of treatment within a single visit to the health facility. In contrast, the SOC typically requires a minimum of two visits: one for submitting the sample, and another for receiving the results. Since the cost data were collected for only one visit in the SOC, we adjusted the costs accordingly. Specifically, we doubled the expenses related to travel, food, childcare and clinic stay for both the patient and caregiver. The cost of lost working days encompasses the total work missed due to illness prior to the first clinical visit, as documented during that visit. This reflects the duration of work absence individuals may experience before receiving a diagnosis. In cases where access to diagnostic services is more challenging, such as when testing is conducted at distant locations or not available onsite, participants are likely to incur greater workday losses before seeking testing. To account for the economic impact of lost workdays up to the point of treatment initiation, we applied a conservative approach by adding the cost of one additional lost workday to the SOC groups in both countries.

#### Health system cost estimation

To generate facility-based diagnostic costs per test across both arms in each setting from a health system perspective, a bottom-up, ingredients-based costing approach was used. In the accompanying CEA study, the analysis was separately for Mozambique and Tanzania; we used average facility-based diagnostic costs of both countries for intervention and standard care arms.

#### Wealth index construction

To categorise the population into different SES groups based on their wealth, measured by assets ownership, a survey was administered including questions about household asset’s ownership during two visits: initial visit and a follow-up at 60 days. These questions were adopted from the well-established Equity Tool, a simple, short and country-specific tool for measuring relative wealth. For Mozambique, the tool included 10 questions, while for Tanzania, 12 questions were used. The complete list of questions for both countries can be accessed through the Equity Tool websites.^19 20^

#### Wealth index analysis

The asset ownership variables were of mixed nature, with approximately half being binary and the rest categorical. To account for this, multiple correspondence analysis was used to generate an asset index for each country, based on the asset ownership data. The first component, whose value exceeds one, was used as the factor weight. Households or participants were then classified into three SES groups (tertile), ranging from the poorest (Q1) to the least poor (Q3), according to the asset index. The wealth indices for both countries were then combined into a single variable for further analysis and distribution is presented in online supplemental appendix table A1.

#### Extended CEA

ECEA provides a framework to assess not only the health impacts and overall costs to policymakers of an intervention but also its broader equity and financial protection implications.^21^ In this study, we applied ECEA components to explore the distributional impact of a decentralised TB diagnostic intervention across SES groups in two countries. We focused on OOP expenditures averted, specifically the additional costs borne by patients in the SOC arm that are reduced under the intervention. This is a key dimension of ECEA, particularly in LMICs where OOP expenditures are a primary driver of financial burden.

To quantify participant costs by SES group k, we estimated the sum of:


$$PC_{k}\;=\left(C_{DM,k}+C_{DNM,k}+C_{IND,k}\right)$$

Here ${PC}_{k}$ is the participant costs incurred by individuals across SES groups (k). $C_{DM,k}$, $C_{DNM,k}$ and $C_{IND,k}$ is the direct medical, direct non-medical and indirect costs incurred per participant for diagnosis of TB across SES groups, respectively.

#### Regression analysis

To evaluate the impact of the intervention on participants’ costs, we also conducted an ordinary least squares regression, since OOP expenditures as the outcome variable is continuous. Since the study design is a randomised controlled trial, adjustment for confounding variables was not required. However, the SES groups are included in the model to confirm the differential effect across SES. To evaluate the distribution of patient costs across SES groups, we calculated concentration indices (CIs) for direct, indirect and total OOP expenditures. The asset-based wealth index was used to rank participants by SES, and CIs were estimated separately for each trial arm. The analysis accounted for individuals with zero expenditures by applying a normalisation method that ensures comparability across the full sample distribution. A positive CI indicates a pro-rich distribution of costs (ie, costs are more concentrated among wealthier participants), while a negative index indicates a pro-poor concentration.

#### Distributional CEA

We calculated the ICER from a societal perspective, incorporating both health system costs and a comprehensive set of participant costs across different SES groups for primary and secondary health outcomes.


$$C_{s,g}=C_{s,g}^{health\;system}+C_{s,g}^{participant}$$

$$ICER_{g}=\frac{C_{1,g}-C_{0,g}}{E_{1,g}-E_{0,g}}$$

where s shows the trial groups, g is the SES groups, C is the cost ($C^{healthsystem}$ is the health system cost and $C^{participant}$ is the participant cost including direct medical, direct non-medical and indirect costs incurred per participant for diagnosis of TB) and E is the health outcomes. Details on the calculation of health system cost are available in a related study from this trial.^17^ In our analysis, we used a health system cost of US$45.5 (95% CI US$40.5 to US$50.5) for the intervention group and US$31 (95% CI US$27.5 to US$33.5) for the control group which is the average of both countries. Health system costs were assumed to be uniform across all SES groups, while OOP expenditures were disaggregated by SES tertile to examine variations in financial burden across socioeconomic groups.

### Role of the funding source

The study’s funding source was not involved in the planning of the investigation, gathering, analysing, interpreting or writing of the report’s contents.

## Results

### Distribution of health outcomes

The numbers and percentages of the health outcomes by SES groups along with breakdowns by country and trial group are presented in table 1. The data reveal relative percentage differences in TB treatment initiation between the intervention (Truenat) and control (SOC) groups across SES groups. In the intervention arm, treatment initiation within both 7 and 60 days is approximately 8% in the poorest group, compared with 5.5% and 5.7%, respectively, in the least poor group. A similar trend is observed in the control group (treatment initiated within 7 days: 6.5% vs 3.5%), although the absolute number of participants initiating treatment within 7 days is notably higher in the intervention group (poorest: 62 vs 34; least poor: 33 vs 25). The detailed table with uncertainty estimates is provided in online supplemental appendix table A7.

**Table 1:** Health outcome by country, trial groups and SES groups

	Total		Mozambique		Tanzania	
	Intervention	SOC	Intervention	SOC	Intervention	SOC
TB treatment initiation within 7 days						
Tertile	N (%)	N (%)	N (%)	N (%)	N (%)	N (%)
Poorest	62 (7.62)	34 (6.54)	32 (7.24)	9 (4.69)	30 (8.06)	25 (7.62)
Middle	52 (8.72)	36 (4.82)	24 (8.19)	14 (4.11)	28 (9.24)	22 (5.42)
Least poor	33 (5.53)	25 (3.51)	18 (5.81)	15 (4.66)	15 (5.23)	10 (2.56)
Total	147 (7.32)	95 (4.8)	74 (7.08)	38 (4.44)	73 (7.59)	57 (5.07)
TB treatment initiation within 60 days						
Poorest	63 (7.74)	48 (9.23)	33 (7.47)	10 (5.21)	30 (8.06)	38 (11.59)
Middle	53 (8.89)	50 (6.69)	25 (8.53)	21 (6.16)	28 (9.24)	29 (7.14)
Least poor	34 (5.7)	47 (6.59)	19 (6.13)	26 (8.07)	15 (5.23)	21 (5.37)
Total	150 (7.47)	145 (7.32)	77 (7.37)	57 (6.67)	73 (7.59)	88 (7.82)

%, percentages; N, number of observations; SOC, standard of care.

In both countries, Mozambique and Tanzania, the intervention group follows a consistent trend. In Mozambique, TB treatment initiation within 7 days is higher among the poorest group (7.2%) compared with the least poor (5.8%), and similarly for treatment within 60 days (7.5% vs 6.1%). In Tanzania, treatment initiation within both 7 and 60 days is also higher in the poorest group (8.1%) compared with the least poor (5.2%). However, the control group in Mozambique does not follow a clear SES gradient.

### Distribution of participants’ costs

Participant direct medical, direct non-medical and indirect costs for the overall sample are presented in table 2, broken down by trial group and SES categories. The average participant cost is higher in the control group (US$13.61) as compared with intervention group (US$8.82). Additionally, the data highlight the cost differences within the SES groups. In the intervention arm, the participant cost for the poorest group is US$8.14, compared with US$10.94 for the least poor group. In the control arm, the least poor group incurs higher cost (US$17.30) compared with the poorest group (US$11.08). A detailed breakdown of the different categories of participant costs is presented in online supplemental appendix table A2 by country.

**Table 2:** Breakdown of different components of average patient costs by trial groups

	Direct cost (US$)		Indirect cost (US$)		Total cost (US$)	
	Mean (95% CI)	Mean (95% CI)	Mean (95% CI)	Mean (95% CI)	Mean (95% CI)	Mean (95% CI)
	Intervention	SOC	Intervention	SOC	Intervention	SOC
Poorest	3.88 (2.89 to 4.86)	4.44 (2.94 to 5.94)	5.86 (3.69 to 8.02)	9.08 (4.87 to 13.29)	8.14 (6.06 to 10.22)	11.08 (7.23 to 14.93)
Middle	3.46 (2.06 to 4.85)	6.26 (3.77 to 8.75)	5.10 (2.50 to 7.70)	8.02 (3.95 to 12.09)	7.49 (4.83 to 10.14)	12.17 (8.14 to 16.20)
Least poor	4.60 (2.55 to 6.64)	11.90 (7.67 to 16.13)	6.74 (3.12 to 10.35)	8.22 (5.54 to 10.90)	10.94 (6.49 to 15.38)	17.30 (12.49 to 22.10)
Concentration index	0.07	0.28	0.07	−0.02	0.08	0.13
P values	0.29	0.00***	0.41	0.84	0.15	0.02**

CI (in parenthesis).

All costs are presented in US dollars.

*p<0.1; **p<0.05; ***p<0.01.

SOC, standard of care; US$, US dollars.

We assessed the degree of inequality among different SES groups using concentration index (CI) measures for all costs categories. The findings show significant inequalities exist in both direct and total costs for the control group (p values <0.05). The CI values suggest that these costs are disproportionately borne by wealthier individuals, with direct costs showing a CI of 0.28 and total costs 0.13, indicating relatively higher relative burden for the least poor group compared with poorest.

A detailed breakdown of overall participant costs, categorised by trial group, is presented in online supplemental table A3. The results highlight notable differences in the average participant costs between the trial groups, showing that the average costs across all categories are higher in the control groups compared with intervention group. Additionally, a comparable breakdown of participant costs for Mozambique and Tanzania is presented in online supplemental appendix table A4. Overall, 10 participants from the SOC group reported borrowing money, while 10 sold property. In the intervention group, 11 participants borrowed money, and 5 sold property.

### Extended CEA

**Figure 1:**
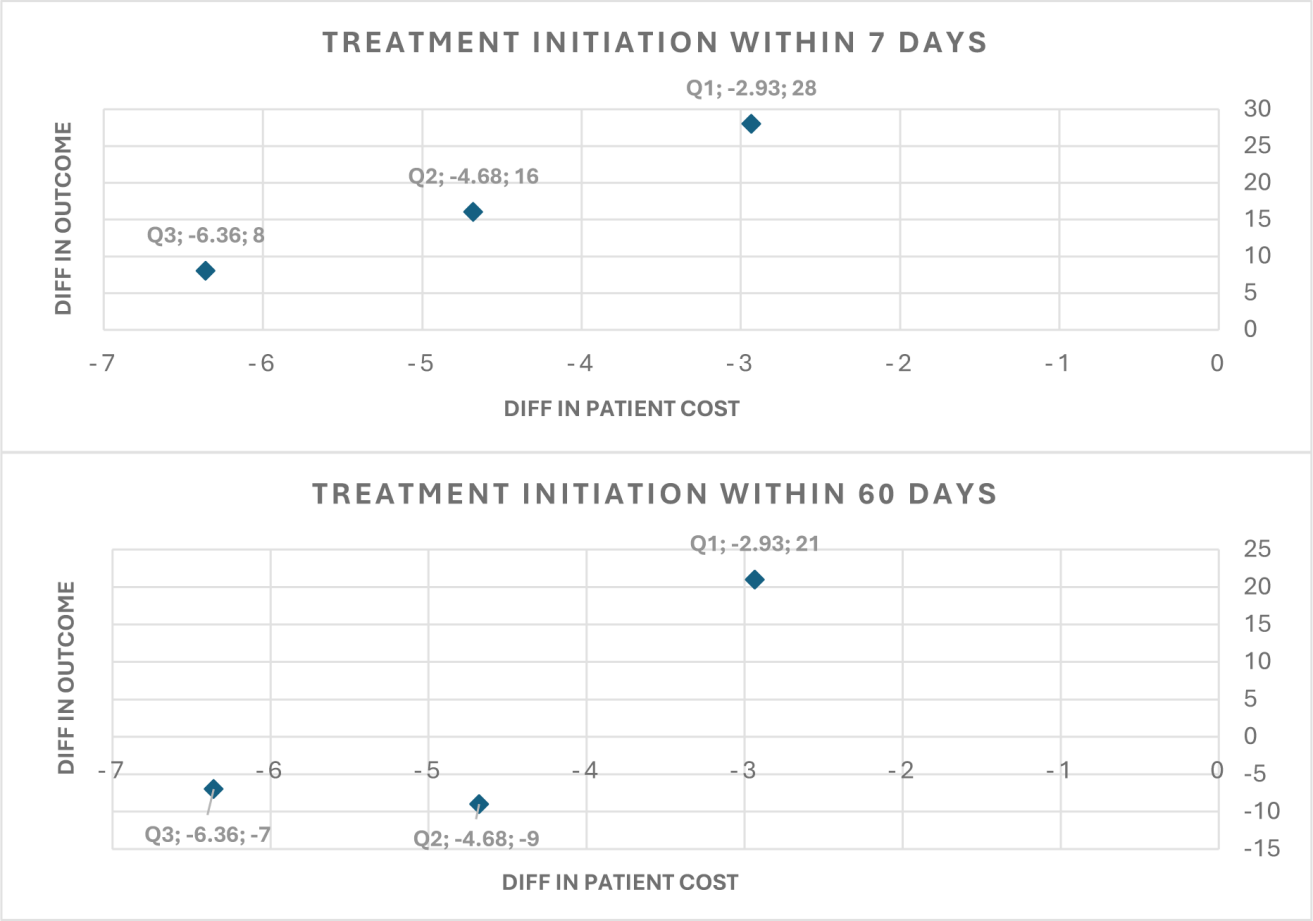
Difference in patient costs and outcome by the socioeconomic status groups. Note: The X-axis shows the difference in the average patients cost in US dollars and the Y-axis shows the difference in the outcome, which is the number of treatment initiations in 7 and 60 days. Q1 represents the poorest group, whereas Q3 represents the least poor group.

We performed the ECEA by comparing the incremental total participant costs with incremental health outcomes (TB treatment initiated at 7 and 60 days). This assessment examines the relationship between additional costs incurred and additional health benefits across all SES groups, and findings are illustrated in figure 1.

The difference in primary health outcome, the absolute number of TB treatment initiated with 7 days, is greatest in the poorest group (Q1: 28) as compared with the least poor group (Q3: 8), while the difference in participant costs is the highest in the least poor group (−US$6.36), which bears the largest costs in both intervention and control groups. This reflects the SES-based variations in participant costs, particularly in the control group. A breakdown of differences in participant costs and health outcomes by country is provided in online supplemental appendix figure A2.

### Regression analysis

The findings of regression analysis reveal that the intervention has a significant negative impact on overall participant costs, suggesting that the participants in the intervention group incur lower total, direct and indirect costs. Total costs were reduced by US$4.63, with a notable US$3.70 reduction in direct costs (p<0.01). Indirect costs also decreased by US$2.52, with marginal statistical significance (p<0.1). Additionally, SES differences play an important role, compared with the poorest group, middle-income participants did not show any significant differences in total, direct or indirect costs, suggesting similar cost burdens. In contrast, least poor participants incurred US$4.58 more in total costs (p<0.05) and US$4.23 more in direct costs (p<0.01), while no significant difference was observed in indirect costs. The results of the regression analysis are presented in online supplemental table A5.

### Distributional CEA

The ICER per additional participant initiating treatment within 7 days of enrolment is US$778 for the poorest group, while for the other two groups, the intervention dominates SOC with higher treatment initiation (52 vs 36 for middle, 33 vs 25 for least poor) at lower costs (table 3). (Although OOP costs are lower for the poorest group, the higher number of poorest patients in the intervention arm (814 vs 520 in SOC (for details on participants’ distribution, please see online supplemental table A1)) inflates total costs when combined with constant system costs. This reflects underlying inequities in how poorest patients access and experience services.) For TB treatment initiation at 60 days, the ICER is US$1452 for the poorest group and US$57 for the least poor group. The ICER for the middle group indicates that, within the 60-day window, few people left to start treatment because most had started before. Online supplemental appendix table A6 provides a detailed breakdown of the ICER for decentralised versus hub-and-spoke TB testing across SES groups by country.

**Table 3:** Incremental cost-effectiveness ratio of decentralised and hub-and-spoke testing for tuberculosis across SES groups

	Intervention		SOC		
	Outcome	Cost (US$)	Outcome	Cost (US$)	ICER*
Outcome: TB treatment initiated with 7 days					
Poorest	62	43 665.45	34	21 880.58	778.03
Middle	52	31 579.52	36	32 244.86	Dominates SOC†
Least poor	33	33 692.42	25	34 434.98	Dominates SOC
Outcome: TB treatment initiated within 60 days					
Poorest	63	43 665.45	48	21 880.58	1452.32
Middle	53	31 579.52	50	32 244.86	Dominates SOC
Least poor	34	33 692.42	47	34 434.98	57.12

* Incremental cost per 7-day treatment initiation and incremental cost per 60-day treatment initiation.

† Intervention is both less costly and more effective as compared with SOC—cost-saving.

ICER, incremental cost-effectiveness ratio; SES, socioeconomic status; SOC, standard of care; TB, tuberculosis.

## Discussion

This paper makes a unique contribution by providing the patient-level comparative analysis of the economic burden of decentralised TB diagnostic services across SES groups in two high-burden countries, Mozambique and Tanzania. By estimating both direct and indirect patient costs, we add a patient-centred perspective to evidence that decentralised testing is effective and cost-effective versus hub-and-spoke models. The findings showed that the decentralised TB testing using the Molbio Truenat platform is cost-saving from the patient perspective. Previous studies have shown that decentralised TB diagnosis is effective and cost-effective compared with a hub-and-spoke testing model.^3 5 22^ Additionally, a recent modelling study also conducted budget impact analysis along with CEA in six high burden countries (Cambodia, Cameroon, Côte d’Ivoire, Mozambique, Sierra Leone and Uganda), concluding that, compared with the SOC, decentralising childhood TB diagnosis to district hospitals could be cost-effective. However, decentralisation would require significant upfront investment.^22^

The findings show that the total OOP expenditures per participant were higher in the SOC arm (US$13.61) compared with the intervention (US$8.82), with similar patterns in Mozambique (US$6.02 vs US$3.82) and Tanzania (US$19.50 vs US$13.25). The average costs were consistently higher in the SOC arm across all SES groups. In contrast, patients in the intervention arm experienced better health outcomes at lower OOP costs, supporting decentralised models that improve access and reduce patient burden.

In our study, average total participant costs were in higher SES groups across both arms, reflecting differences in health-seeking behaviour (eg, private transport, supplementary care, etc) as well as greater ability or willingness to pay for additional services. The least poor incurred higher costs than the poorest (difference US$3.68 in the intervention vs US$5.85 in SOC), aligning with prior literature (Assebe *et al*^13^ and Portnoy *et al*^16^). A recent meta-regression analysis, using data from 22 national surveys, examined the costs faced by the participants with TB, covering both direct and indirect expenses as well as catastrophic costs. The study revealed that the average participant cost was US$2467 (US$2100 to US$2951) for the richest quintile, compared with US$656 (US$496 to US$851) for the poorest.^16^ These patterns suggest that least-poor groups, often more urban or economically stable, engaged more with cost-incurring services and thus benefitted more in absolute monetary terms from decentralisation.

Incremental participant costs indicate greater absolute savings for higher SES groups within the intervention (least poor vs poorest: −US$6.36 vs −US$2.93). This is partly because least poor groups bear the highest costs in both the intervention and control arms, with the cost difference between the two arms being more pronounced for the least poor group. This pattern may be explained by the fact that participants in higher SES groups tend to incur higher baseline costs such as using private transport, purchasing additional services or losing higher-value work time, so they stand to benefit more in absolute terms from the reduced number of visits and faster diagnosis offered by the intervention. As a result, while all groups experience savings, the financial impact is more pronounced among higher SES participants due to their initially higher cost burden. This has implications for policymakers: although the intervention is cost-saving overall, targeted efforts may be needed to ensure the poorest populations can also access and benefit from decentralised services at scale, particularly in more rural or underserved regions.

For the poorest group, the intervention improved treatment initiation within 7 days (28 vs 8) but at slightly higher cost than SOC, yielding an ICER of US$778.03 per additional patient initiated; for middle and least-poor groups, the intervention was dominant. This cost-effectiveness differential across SES groups may be driven by variations in baseline cost exposure, healthcare-seeking behaviour and distribution of participants in each SES group. For the poorest group, while health benefits are clear, small increases in costs relative to their income can be disproportionately burdensome. The ICER for this group was still favourable.

However, previous studies have shown that active case finding and most likely decentralised diagnostic approaches reduce catastrophic costs for participants.^23^,^25^ Taken together, this evidence supports the case for decentralising TB diagnostic services as a strategy not only for improving clinical outcomes but also for alleviating the economic burden on vulnerable populations. This supports the WHO End TB Strategy’s goal of eliminating catastrophic costs for TB-affected families by 2025. Future analyses may also consider applying equity weights to health outcomes or exploring which cost categories (eg, direct medical vs transport vs opportunity cost) most influence ICER variation across SES groups. These refinements can help ensure that equity considerations are better integrated into resource allocation decisions.

Similar benefits have been observed in other health areas; for example, decentralising antiviral therapy services. A study by Abongomera *et al.*^26^ in Malawi and Uganda reported that shifting care to local clinics reduced patient costs (eg, transport, food) and improved outcomes like adherence and retention compared with centralised care.

Our study has several limitations. First, the participant’s cost data were collected based on participant recall, which could introduce recall bias. However, baseline interviews were conducted during participants’ initial visit, when they were more likely to recall their expenses accurately. Second, OOP expenditures were recorded only at the first visit, so we could not capture costs associated with follow-up visits. While we adjusted costs in the SOC arm by doubling certain components, a follow-up cost survey would likely have yielded more accurate estimates. Third, due to 50–60% missing income data, we were unable to calculate catastrophic health expenditures and assess the impoverishment impact of TB diagnostics. Fourth, while the pooled analysis strengthens statistical power, country-specific factors such as healthcare infrastructure or patient behaviour may limit the generalisability of pooled estimates to diverse local health system settings. Despite the limitations of individual country data, both nations are considered high-burden countries and share similar geographical characteristics, making the pooled analysis more meaningful and robust. The findings for each country are presented separately in the appendices. Fifth, aggregating health system costs across countries may obscure meaningful differences in implementation costs. Factors such as staffing models, training intensity, supply chain logistics and supervision arrangements can vary considerably by setting and influence programme costs. Sixth, some imbalance in SES distribution between trial arms reflects facility-level recruitment patterns and, while adjusted for in regression models, may limit direct comparison of unadjusted outcomes across arms. Last, while the Equity Tool offers a practical and standardised method for classifying participants into SES groups, it simplifies the process compared with using the complete Demographic and Health Survey (DHS) asset questionnaire. This approach enables quicker field implementation and comparability across settings but may miss nuances in local socioeconomic variation. Despite these limitations, it remains a useful tool for equity analysis in resource-constrained settings.

This study has several noteworthy strengths. To our knowledge, this is the first study to estimate both direct and indirect patient costs across various SES groups, comparing decentralised TB testing with the standard hub-and-spoke model of care. This analysis highlights the financial implications of different diagnostics approaches and enhances the understanding of the economic burden faced by different SES groups, informing future policy considerations. Future research should further investigate catastrophic costs, the impact of impoverishment with SES disparities, as these findings can guide further investigations and inform policy decisions.

## Conclusion

Although many countries have implemented policies providing free access to TB services, patients in LMICs continue to face a substantial burden of OOP expenses associated with TB care. Decentralised TB testing has the potential to significantly reduce the time to diagnosis and to facilitate faster linkage to treatment, with many patients’ beginning TB treatment within 7 days of diagnosis, while also lowering patient costs. This approach not only improves patient health outcomes but also reduces the economic burden on patients suggesting that the intervention may be especially beneficial for those most vulnerable. These distributional findings underscore the potential of decentralised diagnostics to enhance both equity and effectiveness in TB service delivery.

## Supplementary material

10.1136/bmjgh-2025-020117 online supplemental file 1

## Data Availability

Data may be obtained from a third party and are not publicly available. All data relevant to the study are included in the article or uploaded as supplementary information.
