# Supplementary material for: An extended cost-effectiveness analysis of decentralised TB diagnostic testing with Molbio Truenat MTB/RIF versus hub-and-spoke GeneXpert MTB/RIF in Mozambique and Tanzania
Source: BMJ Glob Health. 2025 Nov 19;10(11):e020117. doi: 10.1136/bmjgh-2025-020117 (PMC12636946; doi:10.1136/bmjgh-2025-020117)
Supplement: online supplemental file 1 [file bmjgh-10-11-s001.docx]

**Supplementary Material**

**Table A1: Asset index distribution**

| Tertials | Mozambique | | | | | | Tanzania | | | | | |
| --- | --- | --- | --- | --- | --- | --- | --- | --- | --- | --- | --- | --- |
|  | Intervention | | SOC | | Total | | Intervention | | SOC | | Total | |
|  | Freq. | Percent | Freq. | Percent | Freq. | Percent | Freq. | Percent | Freq. | Percent | Freq. | Percent |
| Poorest | 442 | 42.3 | 192 | 22.46 | 634 | 33.37 | 372 | 38.67 | 328 | 29.16 | 700 | 33.54 |
| Middle | 293 | 28.04 | 341 | 39.88 | 634 | 33.37 | 303 | 31.50 | 406 | 36.09 | 709 | 33.97 |
| Least poor | 310 | 29.67 | 322 | 37.66 | 632 | 33.26 | 287 | 29.83 | 391 | 34.76 | 678 | 32.49 |
| Total | 1,045 | 100.00 | 855 | 100.00 | 1,900 | 100.00 | 962 | 100.00 | 1,125 | 100.00 | 2,087 | 100.00 |

Note: SOC: standard of care; Freq: frequency

**Figure A1: Health outcomes by trial groups and country**


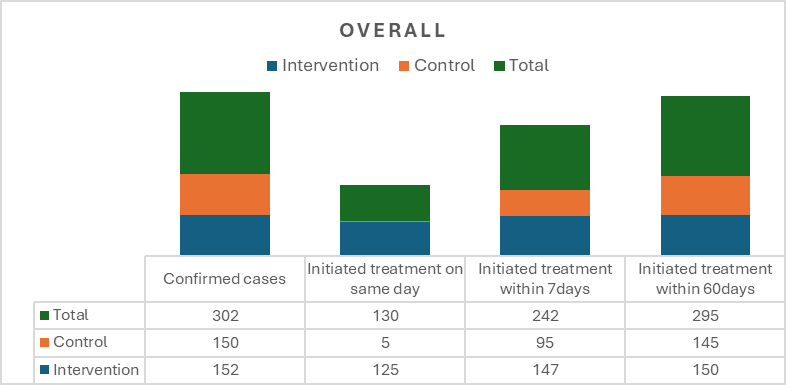

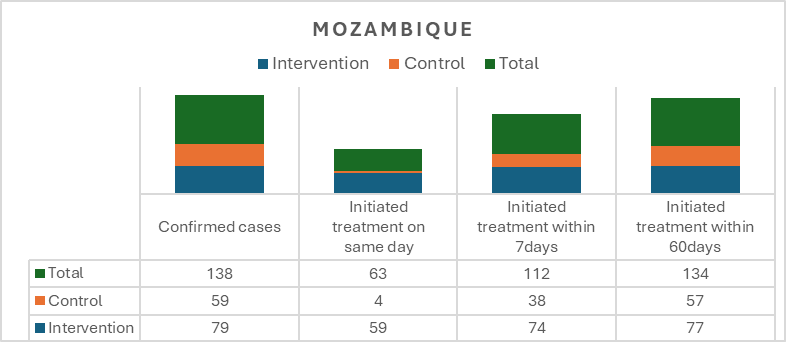

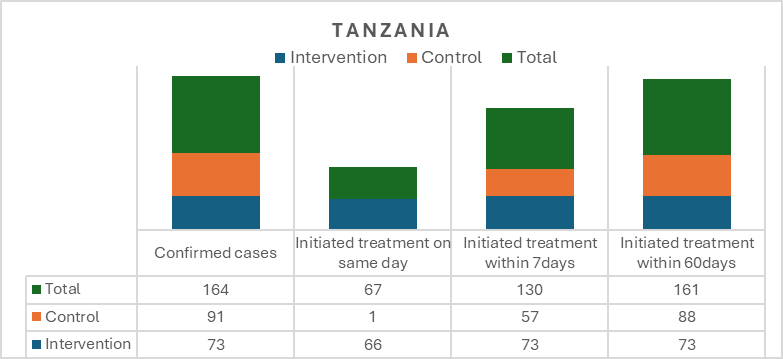


**Table A2:** **Breakdown of different components of average participant costs by country, SES groups and trial groups**

|  | Intervention | | | | | | | | | | SOC | | | | | | | | | | |
| --- | --- | --- | --- | --- | --- | --- | --- | --- | --- | --- | --- | --- | --- | --- | --- | --- | --- | --- | --- | --- | --- |
|  | Direct Cost (USD) | | | Indirect Cost (USD) | | | Total Cost (USD) | | | | Direct Cost (USD) | | | | Indirect Cost (USD) | | | | Total Cost (USD) | | |
|  | Mean | CI (95%) | | Mean | CI (95%) | | Mean | CI (95%) | | Mean | | CI (95%) | | Mean | | CI (95%) | | Mean | | CI (95%) | |
|  | **Mozambique** | | | | | | | | | | | | | | | | | | | | |
| Poorest | 0.74 | 0.10 | 1.37 | 4.89 | 2.25 | 7.54 | 4.22 | 1.92 | 6.51 | 1.04 | | 0.51 | 1.56 | 4.49 | | 0.99 | 7.99 | 4.72 | | 1.67 | 7.77 |
| Middle | 0.94 | 0.20 | 1.67 | 4.53 | 0.64 | 8.43 | 4.16 | 1.16 | 7.17 | 0.59 | | 0.13 | 1.05 | 7.52 | | 2.84 | 12.19 | 7.51 | | 3.15 | 11.86 |
| Least poor | 0.42 | 0.20 | 0.65 | 2.84 | 1.16 | 4.52 | 3.14 | 1.53 | 4.76 | 0.73 | | -0.17 | 1.63 | 5.37 | | 1.65 | 9.08 | 5.60 | | 2.11 | 9.09 |
| Concentration index | -0.19 |  |  | -0.10 |  |  | -0.06 |  |  | -0.14 | |  |  | 0.03 | |  |  | 0.05 | |  |  |
| p-values | 0.12 |  |  | 0.35 |  |  | 0.53 |  |  | 0.28 | |  |  | 0.79 | |  |  | 0.66 | |  |  |
|  | **Tanzania** | | | | | | | | | | | | | | | | | | | | |
| Poorest | 5.45 | 4.39 | 6.51 | 6.45 | 3.26 | 9.64 | 11.26 | 8.33 | 14.20 | 7.70 | | 5.46 | 9.95 | 12.52 | | 5.71 | 19.34 | 15.75 | | 9.81 | 21.70 |
| Middle | 5.32 | 3.18 | 7.47 | 5.47 | 1.79 | 9.15 | 10.21 | 6.16 | 14.26 | 11.93 | | 8.06 | 15.80 | 8.56 | | 1.38 | 15.75 | 16.52 | | 10.01 | 23.04 |
| Least poor | 9.31 | 5.79 | 12.84 | 10.78 | 3.72 | 17.85 | 19.35 | 11.28 | 27.42 | 19.94 | | 14.08 | 25.80 | 10.43 | | 6.66 | 14.21 | 25.30 | | 18.59 | 32.01 |
| Concentration index | 0.12 |  |  | 0.12 |  |  | 0.13 |  |  | 0.25 | |  |  | -0.01 | |  |  | 0.14 | |  |  |
| p-values | 0.03 |  |  | 0.23 |  |  | 0.05 |  |  | 0.00 | |  |  | 0.90 | |  |  | 0.01 | |  |  |

Note: SOC: standard of care, CI: Confidence Intervals, USD: US dollars, all costs are presented in US dollars

**Table A3: Subcategories of overall participant costs by trial groups**

|  |  | **Intervention** | | | | **SOC** | | | |
| --- | --- | --- | --- | --- | --- | --- | --- | --- | --- |
|  |  | N | Mean | N*Mean | SD | N | Mean | N*Mean | SD |
| **Direct medical Cost (USD)** | Consultation fees | 119 | 0.4 | 47.6 | 0.69 | 115 | 0.66 | 75.9 | 0.86 |
|  | Xrays (laboratory) | 96 | 0.48 | 46.08 | 1.55 | 76 | 1.29 | 98.04 | 2.93 |
|  | Medicines | 99 | 0.71 | 70.29 | 1.48 | 86 | 1.09 | 93.74 | 2.19 |
| **Direct non-medical Cost (USD)** | Travel | 129 | 0.74 | 95.46 | 1.16 | 133 | 2.39 | 317.87 | 3.64 |
|  | Food | 111 | 0.53 | 58.83 | 0.85 | 105 | 1.26 | 132.3 | 2.41 |
|  | Childcare | 12 | 0.53 | 6.36 | 0.75 | 8 | 1.7 | 13.6 | 1.98 |
|  | Others | 82 | 0.34 | 27.88 | 0.73 | 80 | 0.45 | 36 | 1.29 |
|  | Caregiver travel | 25 | 0.6 | 15 | 0.72 | 26 | 3.76 | 97.76 | 3.99 |
|  | Caregiver food | 23 | 0.49 | 11.27 | 0.78 | 19 | 2.04 | 38.76 | 2.12 |
|  | Caregiver childcare | 2 | 0 | 0 | 0 | 1 | 0 | 0 | 0 |
| **Indirect cost (USD)** | Clinic stay | 136 | 0.66 | 89.76 | 0.37 | 149 | 1.14 | 169.86 | 0.64 |
|  | Workdays lost | 87 | 9.14 | 795.18 | 11.6 | 75 | 15.26 | 1144.5 | 15.35 |
|  | Borrowed amount | 11 | 9.22 | 101.42 | 8.22 | 10 | 35.93 | 359.3 | 46.4 |
|  | Property value | 5 | 67.94 | 339.7 | 62.12 | 10 | 90.62 | 906.2 | 114.3 |

Note: N: number of observations, SD: standard deviation, SOC: standard of care, USD: US dollars, all costs are presented in US dollars

**Table A4: Subcategories of participant costs by country, and trial groups**

|  |  | Mozambique | | | | Tanzania | | | |
| --- | --- | --- | --- | --- | --- | --- | --- | --- | --- |
|  |  | SOC | | Intervention | | SOC | | Intervention | |
|  |  | Mean | SD | Mean | SD | Mean | SD | Mean | SD |
| Direct medical (USD) | Fees | 0.04 | 0.05 | 0.03 | 0.05 | 1 | 0.9 | 0.67 | 0.8 |
|  | Xraylab | 0.01 | 0.03 | 0 | 0 | 2.08 | 3.51 | 0.81 | 2 |
|  | Medicine | 0.08 | 0.03 | 0.08 | 0.02 | 1.79 | 2.63 | 1.16 | 1.8 |
| Direct non-medical (USD) | Travel | 0.26 | 0.51 | 0.2 | 0.33 | 3.25 | 3.16 | 1.1 | 1.4 |
|  | Food | 0.09 | 0.37 | 0.03 | 0.16 | 1.82 | 2.23 | 0.91 | 1 |
|  | Childcare | --- | --- | 0.53 | 0.91 | 1.27 | 1.48 | 0.53 | 0.8 |
|  | Other | 0.04 | 0.07 | 0.13 | 0.64 | 0.87 | 1.47 | 0.59 | 0.8 |
|  | Caregiver travel | 0.65 | 0.59 | 0.22 | 0.38 | 3.62 | 3.13 | 0.65 | 0.7 |
|  | Caregiver food | 0.59 | 0.99 | 0.79 | 1.58 | 1.96 | 1.65 | 0.43 | 0.6 |
|  | Caregiver childcare | --- | --- | --- | --- | 0 | 0 | 0 | 0 |
| Indirect cost (USD) | Clinic stay | 0.94 | 0.53 | 0.82 | 0.45 | 0.78 | 0.41 | 0.56 | 0.2 |
|  | Workdays lost | 13.54 | 12.1 | 6.31 | 6.46 | 16.1 | 16.8 | 10.9 | 14 |
|  | Borrowed amount | 79.45 | 111 | --- | --- | 25.1 | 18 | 9.22 | 8.2 |
|  | Property value | --- | --- | 80.24 | 110.1 | 90.6 | 114 | 59.8 | 37 |

Note: SD: standard deviation, SOC: standard of care, USD: US dollars, all costs are presented in US dollars

**Figure A2: Incremental participant costs & Outcome by SES groups for Mozambique and Tanzania:**

**(a) Health Outcome: TB treatment initiation within 7 days**


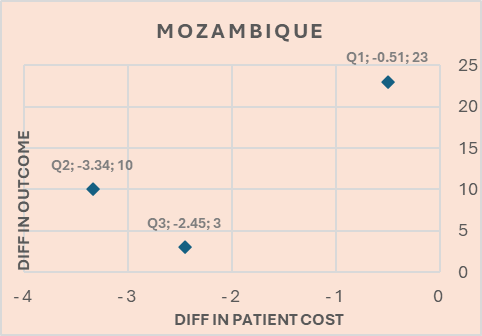

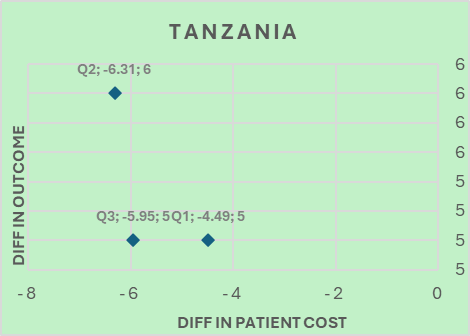


**b) Health Outcome: TB treatment initiation within 60 days**


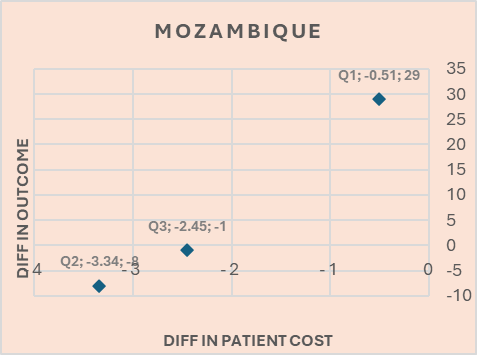

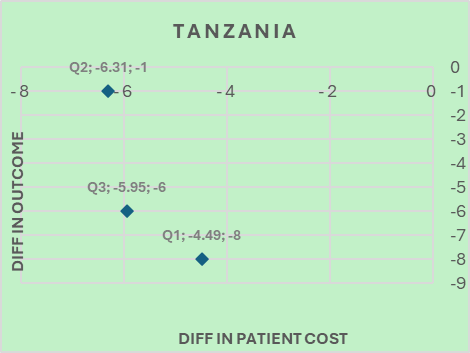


**Table A5: Regression analysis**

|  | Total Cost | Direct Cost | Indirect Cost |
| --- | --- | --- | --- |
| Intervention | -4.632 (1.56) *** | -3.695 (1.03) *** | -2.522 (1.34) * |
| Tertile |  |  |  |
| Middle | 0.203 (1.91) | 0.717 (1.29) | -0.869 (1.64) |
| Least poor | 4.581 (1.89) ** | 4.227 (1.27) *** | 0.012 (1.62) |
| Constant | 11.94 (1.54) *** | 5.974 (1.05) *** | 8.709 (1.35) *** |
| N | 347 | 293 | 305 |
| r2 | 0.0468 | 0.083 | 0.0124 |

Note: *** p<0.01, ** p<0.05, * p<0.1; poorest tertile is considered as base category

**Table A6:** **Breakdown of the ICER for decentralized versus hub-and-spoke tuberculosis testing across SES groups for Mozambique and Tanzania for primary and secondary health outcomes.**

| Outcome: TB Treatment initiated with 7 days | | | | | | Outcome: TB Treatment initiated within 60 days | | | | |
| --- | --- | --- | --- | --- | --- | --- | --- | --- | --- | --- |
|  | Intervention | | SOC | |  | Intervention | | SOC | |  |
|  | Outcome | Cost | Outcome | Cost | ICER | Outcome | Cost | Outcome | Cost | ICER |
|  | **Mozambique** | | | | | | | | | |
| Poorest | 32 | 24405.44 | 9 | 8586.55 | 687.78 | 33 | 24405.44 | 10 | 8586.55 | 687.78 |
| Middle | 24 | 16163.27 | 14 | 16199.68 | Dominates SOC | 25 | 16163.27 | 21 | 16199.68 | Dominates SOC |
| Least poor | 18 | 16784.78 | 15 | 14682.45 | 700.78 | 19 | 16784.78 | 26 | 14682.45 | Dominates SOC |
|  | **Tanzania** | | | | | | | | | |
| Poorest | 30 | 19069.33 | 25 | 12382.62 | 1337.34 | 30 | 19069.33 | 38 | 12382.62 | Dominates SOC |
| Middle | 28 | 15214.78 | 22 | 15640.99 | Dominates SOC | 28 | 15214.78 | 29 | 15640.99 | 426.21 |
| Least poor | 15 | 17033.83 | 10 | 18494.29 | Dominates SOC | 15 | 17033.83 | 21 | 18494.29 | 243.41 |

Note: SOC: standard of care

**Table A7: Health outcome by country, trial groups and SES groups**

|  | **Intervention** | | | | | | **SOC** | | | | | |
| --- | --- | --- | --- | --- | --- | --- | --- | --- | --- | --- | --- | --- |
|  | N | Total | % | SE | LB | UB | N | Total | % | SE | LB | UB |
|  | **Total** | | | | | | | | | | | |
|  | **TB treatment initiation within 7 days** | | | | | | | | | | | |
| Poorest | 67 | 808 | 8.29% | 0.010 | 6.39% | 10.19% | 33 | 530 | 6.23% | 0.010 | 4.17% | 8.28% |
| Middle | 46 | 601 | 7.65% | 0.011 | 5.53% | 9.78% | 38 | 737 | 5.16% | 0.008 | 3.56% | 6.75% |
| Least poor | 34 | 598 | 5.69% | 0.009 | 3.83% | 7.54% | 24 | 713 | 3.37% | 0.007 | 2.04% | 4.69% |
| Total | 147 | 2007 | 7.32% | 0.006 | 6.18% | 8.46% | 95 | 1980 | 4.80% | 0.005 | 3.86% | 5.74% |
|  | **TB treatment initiation within 60 days** | | | | | | | | | | | |
| Poorest | 67 | 808 | 8.29% | 0.010 | 6.39% | 10.19% | 47 | 530 | 8.87% | 0.012 | 6.45% | 11.29% |
| Middle | 48 | 601 | 7.99% | 0.011 | 5.82% | 10.15% | 51 | 737 | 6.92% | 0.009 | 5.09% | 8.75% |
| Least poor | 35 | 598 | 5.85% | 0.010 | 3.97% | 7.73% | 47 | 713 | 6.59% | 0.009 | 4.77% | 8.41% |
| Total | 150 | 2007 | 7.47% | 0.006 | 6.32% | 8.62% | 145 | 1,980 | 7.32% | 0.006 | 6.18% | 8.47% |
|  | **Mozambique** | | | | | | | | | | | |
|  | **TB treatment initiation within 7 days** | | | | | | | | | | | |
| Poorest | 37 | 436 | 8.49% | 0.013 | 5.87% | 11.10% | 8 | 202 | 3.96% | 0.014 | 1.27% | 6.65% |
| Middle | 18 | 298 | 6.04% | 0.014 | 3.34% | 8.75% | 16 | 331 | 4.83% | 0.012 | 2.52% | 7.14% |
| Least poor | 19 | 311 | 6.11% | 0.014 | 3.45% | 8.77% | 14 | 322 | 4.35% | 0.011 | 2.12% | 6.58% |
| Total | 74 | 1,045 | 7.08% | 0.008 | 5.53% | 8.64% | 38 | 855 | 4.44% | 0.007 | 3.06% | 5.83% |
|  | **TB treatment initiation within 60 days** | | | | | | | | | | | |
| Poorest | 37 | 436 | 8.49% | 0.013 | 5.87% | 11.10% | 9 | 202 | 4.46% | 0.015 | 1.61% | 7.30% |
| Middle | 20 | 298 | 6.71% | 0.014 | 3.87% | 9.55% | 22 | 331 | 6.65% | 0.014 | 3.96% | 9.33% |
| Least poor | 20 | 311 | 6.43% | 0.014 | 3.70% | 9.16% | 26 | 322 | 8.07% | 0.015 | 5.10% | 11.05% |
| Total | 77 | 1045 | 7.37% | 0.008 | 5.78% | 8.95% | 57 | 855 | 6.67% | 0.009 | 4.99% | 8.34% |
|  | **Tanzania** | | | | | | | | | | | |
|  | **TB treatment initiation within 7 days** | | | | | | | | | | | |
| Poorest | 30 | 372 | 8.06% | 0.014 | 5.30% | 10.83% | 25 | 328 | 7.62% | 0.015 | 4.75% | 10.49% |
| Middle | 28 | 303 | 9.24% | 0.017 | 5.98% | 12.50% | 22 | 406 | 5.42% | 0.011 | 3.22% | 7.62% |
| Least poor | 15 | 287 | 5.23% | 0.013 | 2.65% | 7.80% | 10 | 391 | 2.56% | 0.008 | 0.99% | 4.12% |
| Total | 73 | 962 | 7.59% | 0.009 | 5.91% | 9.26% | 57 | 1,125 | 5.07% | 0.007 | 3.79% | 6.35% |
|  | **TB treatment initiation within 60 days** | | | | | | | | | | | |
| Poorest | 30 | 372 | 8.06% | 0.014 | 5.30% | 10.83% | 38 | 328 | 11.59% | 0.018 | 8.12% | 15.05% |
| Middle | 28 | 303 | 9.24% | 0.017 | 5.98% | 12.50% | 29 | 406 | 7.14% | 0.013 | 4.64% | 9.65% |
| Least poor | 15 | 287 | 5.23% | 0.013 | 2.65% | 7.80% | 21 | 391 | 5.37% | 0.011 | 3.14% | 7.61% |
| Total | 73 | 962 | 7.59% | 0.009 | 5.91% | 9.26% | 88 | 1125 | 7.82% | 0.008 | 6.25% | 9.39% |

Note: SOC: standard of care; N: number of observations; %: percentages; SE: Standard Error; LB: Lower bound; UB: Upper bound

# Supplementary Material S1: Health system costs

A combination of data collection tools, trial expense reports, and product catalogues were analysed to assess the per test cost component borne by the health system.

## S1.1 Data collection tools

We interviewed study teams, primarily the costing coordinator across the four sites. These coordinators were the primary personnel responsible for completing the implementation costing tools, and for providing qualitative inputs on how the trial was implemented across the clinics in their respective sites.

A set of three tools were developed to assess implementation costs across the study sites.

**S1.1.1 External laboratory implementation costs**

This assessment tool was developed to evaluate the operational characteristics of TB and HIV diagnostics “laboratories” (external laboratories or centralized hospitals) located near the study sites. We aimed to gather detailed information about the laboratories’ processes, costs, and infrastructure, but it was not intended as a performance assessment instrument.

For each type of test, TB or HIV, we aimed to capture details on the testing platform used (e.g., model name) and the number of samples analyzed per month. The sample collection process for both TB sputum and HIV viral load samples was investigated by querying how samples are received from clinics or other collection sites, including the frequency and turnaround time of these collections.

In the laboratory, we aimed to assess total spending on TB sputum and HIV viral load testing over the past month, ideally including an average monthly breakdown of various operational costs. These costs were to include equipment maintenance, technical expertise, building lease, utilities, and staff hours dedicated to leadership and administration. Additionally, the tool queried the cost per test for both TB diagnostics and HIV viral load testing, along with the transportation cost per sample.

We also aimed to explore the detailed steps involved in processing TB sputum and HIV viral load samples. For each testing platform, the steps from initial participant contact to the delivery of results were to be outlined, noting the percentage of specimens lost at each step. The average cost and time for various components, such as cartridge costs, consumables, result delivery, and staff time, were also examined.

Furthermore, we aimed to capture total revenue amassed from participants, clinics, or other facilities for TB sputum and HIV viral load testing in the past month. Finally, information on the laboratory’s operational hours was gathered, including the number of days per week and the average number of hours per day the laboratory operates.

The tool has been presented below in grey font.

**External laboratory (centralized lab or hospital)**

Meet the lab in charge who will guide you to the persons(s) who will best be able to answer these questions.

This assessment form is a tool developed for the TB CAPT study to assess the current operational characteristics of TB and HIV Diagnostics laboratories that are closest to the TB CAPT study sites.

Here, laboratories may mean a.) centralized lab operating machines at high throughput b.) secondary or tertiary hospitals with TB testing capacity.

This tool is not a performance assessment tool. It is an instrument that will help the research team assess how certain practices, from the clinics to the laboratories, can be streamlined to make operational costs more efficient from a societal lens, benefitting both the patients and the health system.

1a. Name of laboratory

1b. Name of town/district

**HIV-TB Diagnostic Infrastructure and Status**

2a. Does the laboratory test TB sputum samples?

i. Yes

ii. No

2b. If yes to 2a, please also mention the platform (model name) and the number of samples analyzed per month

For example if there are four 16-module Xpert Ultra machines in the lab and in the last month 430 sputum samples have been tested then answer as Xpert Ultra 16 module (4) - 430

__________________________________________________________________________________

__________________________________________________________________________________

3a. Does the laboratory test HIV viral load samples?

i. Yes

ii. No

3b. If yes to 2a, please also mention the platform (model name) and the number of samples analyzed per month

For example if there are four 16-module Xpert  machines in the lab and in the last month 430 viral load samples have been tested then answer as Xpert  16 module (4) - 430

_____________________________________________________________________________

____________________________________________________________________________

4a  How do you receive TB sputum samples from a clinic or another site where sample collection takes place. Please mention the frequency in front of the selected option?

For example, staff from clinic may take samples once a week, twice a week, more than once a week or once in two weeks

i. Staff from clinic or other sample collection site bring samples to the laboratory, enter frequency here ____________________________________

ii. Staff from laboratory  collects samples from the clinic, enter frequency here ________________________________

iii. An external courier system or external individual (please specify): ____________________________, enter frequency here ________________________________

iii. Other method (specify) : _____________________________, enter frequency here ___________________________

4b  How do you receive HIV viral load samples from a clinic or another site where sample collection takes place. Please mention the frequency in front of the selected option?

For example, staff from clinic may take samples once a week, twice a week, more than once a week or once in two weeks

i. Staff from clinic or other sample collection site bring samples to the laboratory, enter frequency here ____________________________________

ii. Staff from laboratory  collects samples from the clinic, enter frequency here ________________________________

iii. An external courier system or external individual (please specify): ____________________________, enter frequency here ________________________________

iii. Other method (specify) : _____________________________, enter frequency here ___________________________

5a For each type of test, how much time on an average does it take for a patient to get their TB sputum sample test results, from the day the laboratory received the test to the day the result was reported

Enter test platform ______________________ Enter number of days________________________

Enter test platform ______________________ Enter number of days________________________

Enter test platform ______________________ Enter number of days________________________

Enter test platform ______________________ Enter number of days________________________

Enter test platform ______________________ Enter number of days________________________

Enter test platform ______________________ Enter number of days________________________

5b For each type of test, how much time on an average does it take for a patient to get their HIV viral load test results, from the day the laboratory received the test to the day the result was reported

Enter test platform ______________________ Enter number of days________________________

Enter test platform ______________________ Enter number of days________________________

Enter test platform ______________________ Enter number of days________________________

Enter test platform ______________________ Enter number of days________________________

Enter test platform ______________________ Enter number of days________________________

Enter test platform ______________________ Enter number of days________________________

6 How much did your lab spend in total on TB sputum and HIV viral load testing in the last month ________________________

7. Please provide details on how much would you expect to pay for the following per month

Note: We need an average breakdown below. Numbers need not add up to total costs, but should not exceed total cost

If values available in year, please divide by 12

|  | TB sputum | HIV viral load | Note: |
| --- | --- | --- | --- |
| Average equipment maintenance cost per month |  |  | cost to outside technical experts and/or their own staff time |
| % of time equipment not available per month |  |  | Rough estimate |
| Number of staff hours for leadership/ admin per month |  |  | How many cumulative hours spent to oversee samples |
| Building lease per month |  |  | Proportionally depending on how much of the facility used for TB sputum or HIV viral load testing |
| Utilities and other costs such as security per month |  |  |  |

8a For a single average test performed for TB diagnostics samples please provide the cost per test

Enter test platform ___________________ Enter testing cost ____________________________

Enter test platform ___________________ Enter testing cost ____________________________

Enter test platform ___________________ Enter testing cost ____________________________

8b For a single average test performed for HIV viral load samples please provide the cost per test

Enter test platform ___________________ Enter testing cost ____________________________

Enter test platform ___________________ Enter testing cost ____________________________

Enter test platform ___________________ Enter testing cost ____________________________

9a. For TB diagnostics tests, please mention the sample transportation cost per sample

Note: If costs aggregated by batch, then divide the total costs by the total number of samples per batch

_____________________________________________________________________________

9b. For HIV viral load tests , please mention the sample transportation cost per sample

Note: If costs aggregated by batch, then divide the total costs by the total number of samples per batch

_____________________________________________________________________________

**10a For the TB sputum tests that are performed please provide the following details**

Here platform may be Xpert, Molbio,  or any other dealing with TB sputum samples

1. Enter test platform ________________________________

**For the platform listed above where testing happens at clinic, list out the steps that would be required for processing that specimen, from initial patient contact to returning of results**

**For each step, mention approximately what percentage of specimens are lost**

Step 1:

Step 2:

Step 3:

Step 4:

Step 5:

Step 6:

**For the platform listed above, provide the average cost or time for the following components**

**Cartridge costs**

**Other consumables**

**Cost of result delivery**

**Time from result availability to result delivery**

**Staff member, their level, and hours they spend**

1. Enter test platform ________________________________

**For the platform listed above where testing happens at clinic, list out the steps that would be required for processing that specimen, from initial patient contact to returning of results**

**For each step, mention approximately what percentage of specimens are lost**

Step 1:

Step 2:

Step 3:

Step 4:

Step 5:

Step 6:

**For the platform listed above, provide the average cost or time for the following components**

**Cartridge costs**

**Other consumables**

**Cost of result delivery**

**Time from result availability to result delivery**

**Staff member, their level, and hours they spend**

1. Enter test platform ________________________________

**For the platform listed above where testing happens at clinic, list out the steps that would be required for processing that specimen, from initial patient contact to returning of results**

**For each step, mention approximately what percentage of specimens are lost**

Step 1:

Step 2:

Step 3:

Step 4:

Step 5:

Step 6:

For the platform listed above, provide the average cost or time for the following components

Cartridge costs

Other consumables

Cost of result delivery

Time from result availability to result delivery

Staff member, their level, and hours they spend

**10b For the HIV viral load tests  please provide the following details**

1. Enter test platform ________________________________

**For the platform listed above where testing happens at clinic, list out the steps that would be required for processing that specimen, from initial patient contact to returning of results**

**For each step, mention approximately what percentage of specimens are lost**

Step 1:

Step 2:

Step 3:

Step 4:

Step 5:

Step 6:

**For the platform listed above, provide the average cost or time for the following components**

**Cartridge costs**

**Other consumables**

**Cost of result delivery**

**Time from result availability to result delivery**

**Staff member, their level, and hours they spend**

1. Enter test platform ________________________________

**For the platform listed above where testing happens at clinic, list out the steps that would be required for processing that specimen, from initial patient contact to returning of results**

**For each step, mention approximately what percentage of specimens are lost**

Step 1:

Step 2:

Step 3:

Step 4:

Step 5:

Step 6:

**For the platform listed above, provide the average cost or time for the following components**

**Cartridge costs**

**Other consumables**

**Cost of result delivery**

**Time from result availability to result delivery**

**Staff member, their level, and hours they spend**

1. Enter test platform ________________________________

**For the platform listed above where testing happens at clinic, list out the steps that would be required for processing that specimen, from initial patient contact to returning of results**

**For each step, mention approximately what percentage of specimens are lost**

Step 1:

Step 2:

Step 3:

Step 4:

Step 5:

Step 6:

For the platform listed above, provide the average cost or time for the following components

Cartridge costs

Other consumables

Cost of result delivery

Time from result availability to result delivery

Staff member, their level, and hours they spend

11a  In the past month, how much total money was collected from patient, clinics, or other sample collection facilities testing fee for TB sputum testing? ______________________

11b  In the past month, how much total money was collected from patient, clinics, or other sample collection facilities testing fee for HIV viral load testing? ______________________

12. For how many days a week does your laboratory run? ______________________

13. For how many hours per day on average, does your laboratory run? ______________________________

**S1.1.2 Intervention and control clinic implementation costs**

The tool aimed to gather detailed information about the clinics' diagnostic infrastructure and operational processes. For a subset of clinics, ideally one large and one small across the four sites, we aimed to determine whether the clinic collects sputum samples for TB sputum and HIV Viral load testing and, if so, where the samples are tested, including the platform and the number of samples analysed per month. Details regarding the testing platform and monthly test volume for both in-clinic and external laboratory testing were captured.

Staffing information for TB sputum and HIV viral load testing was gathered, including the number of staff responsible and the cumulative number of hours spent per month on each type of testing. The number of staff responsible for both TB and HIV testing was also captured. Since equipment is not shared in the Truenat arm, the corresponding per-test cost for equipment, its maintenance and warranty, and its handling and operations via program staff is expected to be higher in the Truenat arm compared to the standard of care.

For samples tested outside the clinic, we aimed to capture information on how TB sputum and HIV viral load samples are sent to the testing facility, including the frequency and method of transportation. We aimed to determine the average time it takes for participants to receive their TB sputum and HIV viral load test results, from the day of the test to the day the result was reported, for each test platform used.

Cost information for TB sputum and HIV viral load testing was collected, including total spending in the last month, average monthly costs for equipment maintenance, technical expertise, building lease, utilities, and staff hours dedicated to leadership and administration. Additionally, the cost per test for both TB diagnostics and HIV viral load testing, as well as the sample transportation cost per sample for tests not performed at the clinic, was captured.

Detailed steps involved in processing TB sputum and HIV viral load samples at the clinic were explored. For each testing platform, the steps from initial participant contact to the delivery of results were outlined, noting the percentage of specimens lost at each step. The average cost and time for various components, such as cartridge costs, consumables, result delivery, and staff time, were also examined.

Revenue information from participant testing fees for TB and HIV testing in the past month was captured. The tool also aimed to gather information on equipment and consumables used for the TB CAPT study, including those procured by the TB CAPT Core study and those not procured using TB CAPT Core funding but used for the study. Details on training conducted for clinic staff, including the purpose, attendees, and costs associated with the training, were collected.

Monitoring and supervision costs were also captured, including costs incurred during visits made by the site study team to the clinic. A list of clinic staff involved in the TB CAPT Core Study, along with their roles, activities performed, time spent, and estimated salary, was compiled.

Finally, the tool aimed to gather information on miscellaneous costs, including utilities, rent, safety, and security, as well as other operational and transportation costs not categorized elsewhere.

The tool is presented below in grey font:

**Intervention and Control Clinic Implementation Costs**

First ask the clinic in charge who the best person(s) would be to answer the questions below.

If you do not know the exact answer, use the best estimate along with the source you used or the reasons for your assumption.

**Fill the following form once for each intervention clinic.**

1. Name of clinic

1. **HIV-TB Diagnostic Infrastructure and Status –**

**2a. Does the clinic collect sputum samples for TB testing?**

i. Yes

ii. No

**2b. If yes to 2a, where are the TB sputum samples tested? Please also mention the platform and the number of samples analyzed per month**

*For example, if 20 sputum samples analyzed by Xpert Ultra at a centralized laboratory, and 10 sputum samples tested by Molbio Truenat at the clinic, then answer Xpert ultra (20) for external laboratory, and  Truenat (10) for clinic*

i. At this clinic, please mention platform(s) and number of monthly test per platform here

>Name of platform _____________________

>Number of Samples analysed Per month__________________________

ii. Analyzed offsite at an external laboratory, please mention name of laboratory, platform(s) and number of monthly test per platform here

>Name of  the Laboratory_______________________

> Name of platform(s) _____________________

>Number of Samples analysed Per month__________________________

iii. Other location, please mention here, please mention name of location, platform(s) and number of monthly test per platform

>Name of the Location_______________________

> Name of the platform _____________________

>Number of Samples analysed Per month__________________________

**3a. Does the clinic collect samples for HIV viral load testing?**

i. Yes

ii. No

**3b. If yes to 3a, where are the HIV viral load samples tested? Please also mention the platform and the number of samples analyzed per month**

For example, if 20 HIV viral load samples analyzed by Xpert  at a centralized laboratory, then answer Xpert ultra (20) for external laboratory

i. At this clinic, please mention platform(s) and number of monthly test per platform here

> Name of the platform _____________________

>Number of Samples analysed Per month__________________________

ii. Analyzed offsite at an external laboratory, please mention name of laboratory, platform(s) and number of monthly test per platform here

>Name of  the Laboratory_______________________

> Name of platform(s) _____________________

>Number of Samples analysed Per month__________________________

iii. Other location, please mention here, please mention name of location, platform(s) and number of monthly test per platform

>Name of  the Location_______________________

> Name of platform(s) _____________________

>Number of Samples analysed Per month__________________________

4a. How many staff in your facility are responsible for TB sputum testing. Also mention the cumulative number of hours they spend per month on TB testing

For example, if three staff are involved, with two spending 40 hours per month and the third one spending only 10 hours per month on an average, then answer 3 for staff, and 90 for the number of hours

1. Number of staff ___________________________________
2. Cumulative number of hours ________________________________________

4b. How many staff in your facility are responsible for HIV viral load testing . Also mention the cumulative number of hours they spend per month on TB testing

For example, if three staff are involved, with two spending 40 hours per month and the third one spending only 10 hours per month on an average, then answer 3 for staff, and 90 for the number of hours

1. Number of staff ___________________________________
2. Cumulative number of hours ________________________________________

4c. How many staff in your facility are responsible for both TB and HIV testing (may be the same people as in 5a or 5b)

For example, if three staff are involved, with two spending 40 hours per month and the third one spending only 10 hours per month on an average, then answer 3 for staff, and 90 for the number of hours

1. Number of staff ___________________________________
2. Cumulative number of hours ________________________________________

**5a Ask if TB sputum samples tested outside the clinic --> How do you send TB sputum samples to the testing facility. Please mention the frequency in front of the selected option?**

**For example, staff from clinic may take samples once a week, twice a week, more than once a week or once in two weeks**

i. Staff from clinic takes samples from the testing facility, enter frequency here ____________________________________

ii. Staff from testing facility collects samples from the clinic, enter frequency here ________________________________

iii. An external courier system or external individual (please specify): ___________________________, enter frequency here ________________________________

iv. Other method (specify) : _____________________________,

enter frequency here ___________________________

5b Ask if HIV viral load samples tested outside the clinic --> How do you send HIV viral load samples to the testing facility

For example, staff from clinic may take samples once a week, twice a week, more than once a week or once in two weeks

i. Staff from clinic takes samples from the testing facility, enter frequency here ____________________________________

ii. Staff from testing facility collects samples from the clinic, enter frequency here ________________________________

iii. An external courier system or external individual (please specify): ____________________________, enter frequency here ________________________________

iv. Other method (specify) : _____________________________, enter frequency here ___________________________

6a. For each type of test, how much time on an average does it take for a patient to get their TB sputum test results, from the day of the test to the day the result was reported.

Enter test platform ____________________Enter number of days ________________________

Enter test platform ___________________  Enter number of days ______________________________

Enter test platform ___________________ Enter number of days ______________________________

**6b How much time on an average does it take for a patient to get their HIV viral load test results, from the day of the test to the day the result was reported.**

Enter test platform ____________________Enter number of days ________________________

Enter test platform ___________________  Enter number of days ______________________________

Enter test platform ___________________ Enter number of days ______________________________

7 How much did your site spend in total on TB sputum and HIV viral load testing in the last month ________________________

8   Please provide details on how much would you expect to pay for the following per month

Note: We need an average breakdown below. Numbers need not add up to total costs, but should not exceed total cost

If values available in year, please divide by 12

|  | TB sputum | HIV viral load | Note: |
| --- | --- | --- | --- |
| Average equipment maintenance cost per month |  |  | cost to outside technical experts and/or their own staff time |
| % of time equipment not available per month |  |  | Rough estimate |
| Number of staff hours for leadership/ admin per month |  |  | How many cumulative hours spent to oversee samples |
| Building lease per month |  |  | Proportionally depending on how much of the facility used for TB sputum or HIV viral load testing |
| Utilities and other costs such as security per month |  |  |  |

9a For a single average test performed for TB diagnostics samples please provide the cost per test

Enter test platform ___________________Enter testing cost ________________________________

Enter test platform ___________________Enter testing cost ________________________________

Enter test platform ___________________Enter testing cost ________________________________

9b For a single average test performed for HIV viral load samples please provide the cost per test

Enter test platform ___________________Enter testing cost ________________________________

Enter test platform ___________________Enter testing cost ________________________________

Enter test platform ___________________Enter testing cost ________________________________

10a For TB sputum tests that are not performed at the clinic, please mention the sample transportation cost per sample

Note: If costs aggregated by batch, then divide the total costs by the total number of samples per batch

___________________________________________________________________

10b For HIV viral load tests that are not performed at the clinic, please mention the sample transportation cost per sample

Note: If costs aggregated by batch, then divide the total costs by the total number of samples per batch

___________________________________________________________________________________

**11a For the TB sputum tests that are performed at the clinic (and not outside) please provide the following details.**

Here platform may be Xpert, Molbio, smear, or any other dealing with TB sputum samples

1. Enter test platform ________________________________

**For the platform listed above where testing happens at clinic, list out the steps that would be required for processing that specimen, from initial patient contact to returning of results**

**For each step, mention approximately what percentage of specimens are lost**

Step 1:

Step 2:

Step 3:

Step 4:

Step 5:

Step 6:

**For the platform listed above, provide the average cost or time for the following components**

**Cartridge costs**

**Other consumables**

**Cost of result delivery**

**Time from result availability to result delivery**

**Staff member, their level, and hours they spend**

1. Enter test platform ________________________________

**For the platform listed above where testing happens at clinic, list out the steps that would be required for processing that specimen, from initial patient contact to returning of results**

**For each step, mention approximately what percentage of specimens are lost**

Step 1:

Step 2:

Step 3:

Step 4:

Step 5:

Step 6:

**For the platform listed above, provide the average cost or time for the following components**

**Cartridge costs**

**Other consumables**

**Cost of result delivery**

**Time from result availability to result delivery**

**Staff member, their level, and hours they spend**

1. Enter test platform ________________________________

**For the platform listed above where testing happens at clinic, list out the steps that would be required for processing that specimen, from initial patient contact to returning of results**

**For each step, mention approximately what percentage of specimens are lost**

Step 1:

Step 2:

Step 3:

Step 4:

Step 5:

Step 6:

**For the platform listed above, provide the average cost or time for the following components**

**Cartridge costs**

**Other consumables**

**Cost of result delivery**

**Time from result availability to result delivery**

**Staff member, their level, and hours they spend**

**11b For the HIV viral load tests that are performed at the clinic (and not outside) please provide the following details.**

Here platform may be Xpert, Molbio, smear, or any other dealing with TB sputum samples

1. Enter test platform ________________________________

**For the platform listed above where testing happens at clinic, list out the steps that would be required for processing that specimen, from initial patient contact to returning of results**

**For each step, mention approximately what percentage of specimens are lost**

Step 1:

Step 2:

Step 3:

Step 4:

Step 5:

Step 6:

**For the platform listed above, provide the average cost or time for the following components**

**Cartridge costs**

**Other consumables**

**Cost of result delivery**

**Time from result availability to result delivery**

**Staff member, their level, and hours they spend**

1. Enter test platform ________________________________

**For the platform listed above where testing happens at clinic, list out the steps that would be required for processing that specimen, from initial patient contact to returning of results**

**For each step, mention approximately what percentage of specimens are lost**

Step 1:

Step 2:

Step 3:

Step 4:

Step 5:

Step 6:

**For the platform listed above, provide the average cost or time for the following components**

**Cartridge costs**

**Other consumables**

**Cost of result delivery**

**Time from result availability to result delivery**

**Staff member, their level, and hours they spend**

Enter test platform ________________________________

**For the platform listed above where testing happens at clinic, list out the steps that would be required for processing that specimen, from initial patient contact to returning of results**

**For each step, mention approximately what percentage of specimens are lost**

Step 1:

Step 2:

Step 3:

Step 4:

Step 5:

Step 6:

**For the platform listed above, provide the average cost or time for the following components**

**Cartridge costs**

**Other consumables**

**Cost of result delivery**

**Time from result availability to result delivery**

**Staff member, their level, and hours they spend**

12a  In the past month, how much total money was collected from patient testing fee for TB testing? ______________________

12b In the past month, how much total money was collected from patient testing fee for HIV testing? ________________________________

1. **Equipment and consumables that are used for the TB CAPT study**

Prepare a list of equipment used at the clinic procured by the TB CAPT Core study

This may include medical equipment such as the TrueNat TB assay, or the Molbio Truenat platform, sputum cups, etc. Or non medical equipment like laptops, filing cabinet, internet router etc.

Consumables may include test cartridges, reagents, etc.

| List the quantity, unit and total cost (including fee and taxes) |
| --- |
| If known, show service and maintenance costs as a separate line item |
|  |
| *Example: If 100 cartridges are procured by the clinic of which 40 are used for the TB CAPT study, enter 40% under % used for TB CAPT* |

| Equipment | Unit cost | Quantity | Total Cost | % Used for TB CAPT core activities | Source | |
| --- | --- | --- | --- | --- | --- | --- |
|  |  |  |  |  |  | |
|  |  |  |  |  |  |  |
|  |  |  |  |  |  |  |
|  |  |  |  |  |  | |
|  |  |  |  |  |  | |
|  |  |  |  |  |  | |
|  |  |  |  |  |  | |
| Consumables | Unit cost | Quantity used per month | Total Cost | % Used for TB CAPT core activities | Source | |
|  |  |  |  |  |  | |
|  |  |  |  |  |  | |
|  |  |  |  |  |  |  |
|  |  |  |  |  |  |  |
|  |  |  |  |  |  |  |
|  |  |  |  |  |  | |
|  |  |  |  |  |  | |
|  |  |  |  |  |  | |

2.       Were there any delays in procurement of the above listed equipment and consumables?

If yes, what were the reasons for the delays (customs, COVID-19 related delays)? Were there any other challenges faced, e.g. approval of equipment purchase

| Equipment /consumables | Reasons for delay | Duration of delay |
| --- | --- | --- |
|  |  |  |
|  |  |  |
|  |  |  |
|  |  |  |
|  |  |  |
|  |  |  |
|  |  |  |
|  |  |  |
|  |  |  |
|  |  |  |
|  |  |  |
|  |  |  |
|  |  |  |
|  |  |  |
|  |  |  |

3.       Please make a list of all equipment and consumables not procured using TB CAPT Core funding but used for the TB CAPT Core study.

Enter the  name, quantity, year when purchased (if available) and unit and total cost (if available)

| Equipment | | | Unit cost | Quantity | Total Cost | % Used for TB CAPT core activities | Source |
| --- | --- | --- | --- | --- | --- | --- | --- |
|  | | |  |  |  |  |  |
|  | | |  |  |  |  |  |
|  |  |  |  |  |  |  |  |
|  |  |  |  |  |  |  |  |
|  |  |  |  |  |  |  |  |
| Consumables | | | Unit cost | Quantity used per month | Total Cost | % Used for TB CAPT core activities | Source |
|  |  |  |  |  |  |  |  |
|  |  |  |  |  |  |  |  |
|  |  |  |  |  |  |  |  |
|  |  |  |  |  |  |  |  |
|  |  |  |  |  |  |  |  |

iii. **Training conducted for clinic staff**

1.       List out the trainings that have been attended by your clinic along with the purpose for each.

List the team responsible for training, the team attending the training and the number of people at each skill level, and the format of the as given in the example below:

| Training Purpose | Month/Year | Attendees (number) | Format | Curriculum Development | Coordinator |
| --- | --- | --- | --- | --- | --- |
|  |  |  |  |  |  |
|  |  |  |  |  |  |
|  |  |  |  |  |  |
|  |  |  |  |  |  |
|  |  |  |  |  |  |
|  |  |  |  |  |  |
|  |  |  |  |  |  |

| 2.       List any equipment procured or expenses incurred linked to the trainings. |
| --- |
| Please make sure these equipment are not listed in the equipment and consumables section above. Mention the unit cost (including taxes and fees), quantitiy, and total cost |
| Also list the source - e.g. procurement log, expense receipt, finance records, etc. |
| Some examples may include per diem, stationery, room and projector rental, transport, meals, trainer fee, etc. |

| Training Equipment /item | | | Unit cost | Quantity | Total Cost | Source |
| --- | --- | --- | --- | --- | --- | --- |
|  | | |  |  |  |  |
|  | | |  |  |  |  |
|  |  |  |  |  |  |  |
|  |  |  |  |  |  |  |
|  |  |  |  |  |  |  |
|  |  |  |  |  |  |  |
|  |  |  |  |  |  |  |
|  |  |  |  |  |  |  |
|  |  |  |  |  |  |  |
|  |  |  |  |  |  |  |
|  |  |  |  |  |  |  |

**IV. Monitoring and supervision costs**

1.       Apart from introductory visits and trainings, list the visits made by the site study team at this clinic E.g., regulatory visits, delivery of materials, etc.

For each visit, list the corresponding costs incurred

Examples of cost may include per diem, transport, meals, etc.

One row for each cost item. For multiple cost items per visit we will use multiple rows

| Visit Month/Year | Visit Type | Cost Item | Cost incurred |
| --- | --- | --- | --- |
|  |  |  |  |
|  |  |  |  |
|  |  |  |  |
|  |  |  |  |
|  |  |  |  |
|  |  |  |  |
|  |  |  |  |
|  |  |  |  |

V. **Clinic costs and admin**

1.       Provide a list of the names of all clinic staff involved in the TB CAPT Core Study.

2.       For each staff member, list the TB CAPT core study activity conducted by them and the estimated time spent on each activity in a typical week

Note: Use one row per activity. So if staff member has done 3 activities, use 3 rows for that staff member

| Staff Name or Staff No | Role/Title | Activities Performed | Time Spent | Estimated saalry based on Government salary scale |
| --- | --- | --- | --- | --- |
|  |  |  |  |  |
|  |  |  |  |  |
|  |  |  |  |  |
|  |  |  |  |  |
|  |  |  |  |  |
|  |  |  |  |  |
|  |  |  |  |  |
|  |  |  |  |  |
|  |  |  |  |  |
|  |  |  |  |  |
|  |  |  |  |  |

VI. **Miscellaneous costs**

1. List the cost paid for utilities including electricity, internet, etc. and other operational costs such as rent, safety and security that is incurred for the study.

If not available by utility item, list the aggregate utility costs or aggregate operational expenses for running the clinic if available

| Operational cost item | Total Cost | Duration | % Used for TB CAPT core activities | Source |
| --- | --- | --- | --- | --- |
|  |  |  |  |  |
|  |  |  |  |  |
|  |  |  |  |  |

2.       List other miscellaneous costs part of the implementation that are not categorized before.

E.g., transportation costs not linked to training and site visits, etc

| Other item | Total Cost | Duration | % Used for TB CAPT core activities | Source |
| --- | --- | --- | --- | --- |
|  |  |  |  |  |
|  |  |  |  |  |
|  |  |  |  |  |

**S1.1.3 General Questions for study sites: Implementation Costs**

This tool aimed to gather best estimates on several key aspects of trial implementation from the perspective of the central office of each site. The tool aimed to capture information on:

1. Introductory Visits and Site Finalization: This includes documenting the timeline of initial clinic selection, roles of central team members involved, and any challenges causing delays in clinic finalization.
2. Equipment and Consumables: Detailed information on procurement of equipment and consumables, including quantities, unit costs, total costs, and reasons for any procurement delays.
3. Training Conducted for Each Site: Documentation of training sessions attended, purpose of each training, responsible teams, attendees' skill levels, and associated costs not already accounted for in equipment procurement.
4. Main Office Costs and Admin: Listing all staff involved in the trial, their roles, specific trial activities performed, and estimated time spent on each activity.
5. Miscellaneous Costs: Including operational expenses such as utilities, rent, and security, as well as other miscellaneous costs not covered elsewhere in the tool.

The tool has been presented below in grey font.

**General Questions – Site HQ**

For the economic evaluation of the TB CAPT Core trial, we would like to understand the resources, and corresponding costs, required by the respective clinics which are a part of the study, and the corresponding laboratories that serve these clinics.

In addition, we would like to understand the upfront time investment in kickstarting the trial for your respective site.

If you do not know the exact answer, use the best estimate along with the source you used or the reasons for your assumption.

**I.     Introductory visits and site finalization**

| **1.** When did the initial clinic selection process occur? | | | |
| --- | --- | --- | --- |
| **This includes time spent on identifying clinics, gauging interest of clinic participation, preparing and finalizing Memorandum of Understanding and subcontracts with clinics, if any.** | | | |
| **a.** Mention start and end month/year. | | | |
| **b.** List the roles/titles of central team members involved | | | |
| Clinic Name | Start (Month/Year) | End (Month/Year) | Central Team Members Involved |
|  |  |  |  |
|  |  |  |  |
|  |  |  |  |
|  |  |  |  |
|  |  |  |  |
|  |  |  |  |
|  |  |  |  |
|  |  |  |  |
|  |  |  |  |

2.       Were there any challenges faced by the central team that caused delays in the clinic finalization process?

If yes, indicate the reason for the delay (e.g. COVID-19 related delays) and also indicate by how many weeks/months was the clinic finalization delayed as a result.

| Clinic Name | Reasons for delay | Duration of delay |
| --- | --- | --- |
|  |  |  |
|  |  |  |
|  |  |  |
|  |  |  |
|  |  |  |
|  |  |  |
|  |  |  |

3.       Were there any challenges faced by the central team that caused delays in the overall trial start date?

If yes, indicate the reason for the delay (e.g. COVID-19 related delays) and also indicate by how many weeks/months was the trial start delayed as a result.

| Reasons for delay | Duration of delay |
| --- | --- |
|  |  |
|  |  |
|  |  |
|  |  |

**II.                    Equipment and Consumables**

**1.** For each item procured for the central site office or the site headquarters, indicate the quantity, unit cost (including taxes and fee), total cost (including taxes and fee).

Please also list the source for each - e.g. procurement logs, finance records, etc.

Please note: equipment also includes furniture or other tangible items

| Equipment /item | | | Unit cost | Quantity | Total Cost | % Used for TB CAPT core activities | Source |
| --- | --- | --- | --- | --- | --- | --- | --- |
|  | | |  |  |  |  |  |
|  | | |  |  |  |  |  |
|  |  |  |  |  |  |  |  |
|  |  |  |  |  |  |  |  |
|  |  |  |  |  |  |  |  |
|  |  |  |  |  |  |  |  |
|  |  |  |  |  |  |  |  |
|  |  |  |  |  |  |  |  |
|  |  |  |  |  |  |  |  |
|  |  |  |  |  |  |  |  |
|  |  |  |  |  |  |  |  |
|  |  |  |  |  |  |  |  |

2.       Were there any delays in procurement of the above listed equipment and consumables?

If yes, what were the reasons for the delays (customs, COVID-19 related delays)? Were there any other challenges faced, e.g. approval of equipment purchase

| Equipment /item | Reasons for delay | Duration of delay |
| --- | --- | --- |
|  |  |  |
|  |  |  |
|  |  |  |
|  |  |  |
|  |  |  |
|  |  |  |
|  |  |  |
|  |  |  |
|  |  |  |
|  |  |  |

3.       Please make a list of all equipment not procured using TB CAPT Core funding but used for the TB CAPT Core study.

Enter the equipment name, quantity, year when purchased (if available) and unit and total cost (if available)

| Equipment /item | | | Unit cost | Quantity | Total Cost | % Used for TB CAPT core activities | Source |
| --- | --- | --- | --- | --- | --- | --- | --- |
|  | | |  |  |  |  |  |
|  | | |  |  |  |  |  |
|  |  |  |  |  |  |  |  |
|  |  |  |  |  |  |  |  |
|  |  |  |  |  |  |  |  |
|  |  |  |  |  |  |  |  |
|  |  |  |  |  |  |  |  |
|  |  |  |  |  |  |  |  |
|  |  |  |  |  |  |  |  |
|  |  |  |  |  |  |  |  |
|  |  |  |  |  |  |  |  |

**III.  Training conducted for members of each site**

1.       List out the trainings that have been attended by your site along with the purpose for each.

List the team responsible for training, the team attending the training and the number of people at each skill level, and the format of the as given in the example below:

| Training Purpose | Month/Year | Attendees (number) | Format | Curriculum Development | Coordinator |
| --- | --- | --- | --- | --- | --- |
|  |  |  |  |  |  |
|  |  |  |  |  |  |
|  |  |  |  |  |  |
|  |  |  |  |  |  |
|  |  |  |  |  |  |
|  |  |  |  |  |  |

2.       List any equipment procured or expenses incurred linked to the trainings.

Please make sure these equipment are not listed in the equipment and consumables section above. Mention the unit cost (including taxes and fees), quantity, and total cost

Also list the source - e.g. procurement log, expense receipt, finance records, etc.

Some examples may include per diem, stationery, room and projector rental, transport, meals, trainer fee, etc.

| Training Equipment /item | | | Unit cost | Quantity | Total Cost | Source |
| --- | --- | --- | --- | --- | --- | --- |
|  | | |  |  |  |  |
|  | | |  |  |  |  |
|  |  |  |  |  |  |  |
|  |  |  |  |  |  |  |
|  |  |  |  |  |  |  |
|  |  |  |  |  |  |  |
|  |  |  |  |  |  |  |
|  |  |  |  |  |  |  |
|  |  |  |  |  |  |  |
|  |  |  |  |  |  |  |
|  |  |  |  |  |  |  |
|  |  |  |  |  |  |  |

**IV.                    Main Office Costs and Admin**

1.       Provide a list of the names of all central and field staff involved in the TB CAPT Core Study.

2.       For each staff member, list the TB CAPT core study activity conducted by them and the estimated time spent on each activity in days/weeks/months

Note: Use one row per activity. So if staff member has done 3 activities, use 3 rows for that staff member

| Staff Name | Role/Title | Activities Performed | Time Spent |
| --- | --- | --- | --- |
|  |  |  |  |
|  |  |  |  |
|  |  |  |  |
|  |  |  |  |
|  |  |  |  |
|  |  |  |  |
|  |  |  |  |
|  |  |  |  |
|  |  |  |  |
|  |  |  |  |
|  |  |  |  |

**IV.                    Miscellaneous**

1.       List the cost paid for utilities including electricity, internet, etc. and other operational costs such as rent, safety and security that is incurred for the study.

If not available by utility item, list the aggregate utility costs or aggregate operational expenses for running the site HQ if available

| Operational cost item | Total Cost | Duration | % Used for TB CAPT core activities | Source |
| --- | --- | --- | --- | --- |
|  |  |  |  |  |
|  |  |  |  |  |
|  |  |  |  |  |

2.       List other miscellaneous costs part of the implementation that are not categorized before.

E.g., IRB related costs, transportation costs not linked to training and site visits, etc

| Other item | Total Cost | Duration | % Used for TB CAPT core activities | Source |
| --- | --- | --- | --- | --- |
|  |  |  |  |  |
|  |  |  |  |  |
|  |  |  |  |  |

## S1.2 Product Catalogues

To triangulate the prices of testing equipment and consumables, we referred to the Global Drug Facility’s (GDF) product catalogue 2022.

## S1.3 Data analysis

For each of the clinics where data was collected, we recorded the monthly costs and per test costs (where possible to record) as separate line items and tagged them as either consumables, equipment, monitoring & evaluation and communication, staffing, testing, training, warranty & maintenance, and miscellaneous. The monthly costs were then divided by the estimated number of persons tested each month to get the contribution of that specific cost item to the total cost per test. The table below lists the various cost items reported by the clinics, how they were categorized, and how they were captured calculated.

**Table S1**

| Cost category | Cost items included | Calculation |
| --- | --- | --- |
| Consumables | Sample prep kit, pre treatment pack | Per test |
|  | Medical consumables | Captured either in an aggregate manner monthly, or linked to each test |
|  | Reagents | Per test |
| Testing (also a consumable) | cartridge | Per test |
| Equipment | Truenat machine | Annualized assuming an expected life of 10 years and then divided by 12 to determine monthly costs. |
|  | Furniture | Annualized assuming an expected life of 10 years and then divided by 12 to determine monthly costs. |
|  | GX machine 4 module | Annualized assuming a expected life of 10 years and then divided by 12 to determine monthly costs. |
|  | Electrical equipment | Annualized assuming a expected life of 5 years and then divided by 12 to determine monthly costs. |
|  | Medical equipment | Annualized assuming a expected life of 10 years and then divided by 12 to determine monthly costs. |
| M&E and communication | Internal and external monitoring | Monthly |
|  | Internet and communication | Monthly |
|  | Transportation for study monitoring | Monthly |
|  | Supervision | Monthly |
| Staffing | Salaries | Monthly |
| Training | SIV core training | Training costs averaged across participating clinics. Assumed training every 2 years. |
|  | Training materials | Monthly: Hardware with an estimated life of 10 years whereas stationery with a life of 2 years. Certain costs attributed per test. |
|  | Per diems, conference room, and transport | Training costs averaged across participating clinics. Assumed training every 2 years. |
| Warranty and Maintenance | Maintenance | Monthly |
|  | Truenat warranty | Monthly |
|  | GeneXpert warranty | Monthly |
| Miscellaneous | Sample Transport | Per test |
|  | Stationery | Monthly |
|  | IRB/ethics | Monthly: IRB is a one time activity, assuming impact lasts for 10 years |
|  | Results Delivery | Per sample |

In both Tanzania and Mozambique, the average number of tests conducted per month per clinic ranged from 14.8 to 15.7. For the deterministic calculation of the cost per participant tested, we standardized this value to 16 tests across all arms to maintain consistency. This figure was then adjusted by 25% to establish minimum and maximum test numbers per month, resulting in 12 and 18 tests, respectively. In Table S2, "PE" represents the point estimate of each cost component per participant tested. Data were collected from 19 clinics across the four sites, reported either monthly or per sample (as detailed in Table 1 above). Monthly costs were converted to per-sample costs by dividing the monthly costs by the number of samples per month, thereby determining the point estimate cost by category. The maximum and minimum values for each cost category in both countries were derived from the range of values reported by the clinics within the respective countries.

**Table S2**

|  | TANZANIA | | | MOZAMBIQUE | | |
| --- | --- | --- | --- | --- | --- | --- |
|  | PE | Min | Max | PE | Min | Max |
| Testing | 7.90 | 7.89 | 7.91 | 8.95 | 7.89 | 10.01 |
| Consumables | 3.75 | 3.00 | 5.41 | 7.00 | 3.00 | 11.00 |
| Equipment | 10.57 | 7.66 | 15.11 | 9.64 | 7.15 | 13.78 |
| Warranty and maintenance | 6.37 | 5.10 | 8.49 | 6.37 | 5.10 | 8.49 |
| Training | 1.53 | 0.00 | 4.53 | 0.23 | 0.05 | 0.50 |
| M&E and comm | 2.27 | 0.77 | 6.89 | 5.71 | 3.46 | 9.44 |
| Staffing | 4.81 | 0.00 | 13.42 | 10.67 | 6.89 | 18.71 |
| Miscellaneous | 1.64 | 0.06 | 3.75 | 1.41 | 0.25 | 2.66 |
| COST PER PARTICIPANT TESTED | 59.10 | 37.52 | 92.10 | 49.97 | 33.80 | 74.60 |
|  |  |  |  |  |  |  |
|  | TANZANIA | | | MOZAMBIQUE | | |
|  | PE | Min | Max | PE | Min | Max |
| Testing | 9.98 | 9.97 | 9.99 | 9.98 | 9.97 | 9.99 |
| Consumables | 1.67 | 0.00 | 3.47 | 13.35 | 10.02 | 20.02 |
| Equipment | 2.42 | 1.40 | 4.39 | 1.63 | 0.98 | 2.44 |
| Warranty and maintenance | 1.26 | 1.01 | 1.68 | 1.26 | 1.01 | 1.68 |
| Training | 0.91 | 0.00 | 2.41 | 0.24 | 0.05 | 0.47 |
| M&E and comm | 1.75 | 0.90 | 3.22 | 6.08 | 4.47 | 9.44 |
| Staffing | 0.96 | 0.00 | 2.68 | 4.52 | 2.76 | 8.89 |
| Miscellaneous | 2.31 | 0.34 | 5.49 | 1.77 | 0.24 | 4.66 |
| COST PER PARTICIPANT TESTED | 21.26 | 13.61 | 33.35 | 38.83 | 29.49 | 57.59 |

**S1.3.1 Number of participants tested and number of tests**

To calculate the facility based diagnostic cost per participant tested, we took the number of persons tested as the denominator for the calculation of the point estimate. A person may have multiple tests (if one of the tests is invalid), or may have different tests (Xpert/Truenat only or smear only or combination of smear+Xpert/Truenat or any other combination), both of which will impact the true cost per test. To address this, we added uncertainty to the number of people tested (­+ 25% variation), so that the range of values also encompass the actual number of tests. Across both scenarios, more than 98% of the participants received their test results via Xpert (in the standard of care) or Truenat (in the intervention arm).
